# Inventory model for green products with payment strategy, selling price and green level dependent demand using teaching learning based optimization algorithm

**DOI:** 10.1038/s41598-024-53109-w

**Published:** 2024-02-06

**Authors:** Subhash Chandra Das, Hachen Ali, Md. Al-Amin Khan, Ali Akbar Shaikh, Adel Fahad Alrasheedi

**Affiliations:** 1 Department of Mathematics, Chandrapur College, Chandrapur, West Bengal India; 2 Department of Mathematics, The University of Burdwan, Burdwan, 713104 India; 3 Department of Mathematics, Jahangirnagar University, Savar, Dhaka, 1342 Bangladesh; 4 Tecnológico de Monterrey, School of Engineering and Sciences, Ave. Eugenio Garza Sada 2501, 64849 Monterrey, N. L. México; 5 Department of Statistics and Operations Research, College of Science, King Saud University, P.O. Box 2455, 11451 Riyadh, Saudi Arabia

**Keywords:** Engineering, Mathematics and computing

## Abstract

There has been a lot of research on pricing and lot-sizing practices for different payment methods; however, the majority has focused on the buyer’s perspective. While accepting buyers’ credit conditions positively impacts sales, requesting advance payments from purchasers tends to have a negative effect. Additionally, requiring a down payment has been found to generate interest revenue for the supplier without introducing default risk. However, extending the credit period, along with offering delayed payment options, has the potential to increase sales volume, albeit with an elevated risk of defaults. Taking these payment schemes into account, this study investigates and compares the per-unit profit for sellers across three distinct payment methods: advance payment, cash payment, and credit payment. The consumption rate of the product varies non-linearly not only with the time duration of different payment options but also with the price and the level of greenness of the product. The utmost objective of this work is to determine the optimal duration associated with payment schemes, selling price, green level, and replenishment period to maximize the seller’s profit. The Teaching Learning Based Optimization Algorithm (TLBOA) is applied to address and solve three numerical examples, each corresponding to a distinct scenario of the considered payment schemes. Sensitivity analyses confirm that the seller’s profit is markedly influenced by the environmental sustainability level of the product. Furthermore, the seller’s profitability is more significantly affected by the selling price index compared to the indices of the payment scheme duration and the green level in the demand structure.

## Introduction

The primary objective of the retail industry is to receive payment promptly upon product delivery, either in cash or through cash-on-delivery arrangements. Recent polls indicate that a significant majority of American consumers, over 90%, prefer interest-free borrowing within a specific timeframe, commonly known as the ‘buy now, pay later’ option. If a customer pays in full during the credit period, typically, no interest fees are incurred. However, if the purchaser fails to do so, the seller may choose to impose interest on the remaining unpaid amount. Providing credit instead of cash results in reduced holding costs for customers, contributing to an increase in sales volume for the seller. One primary concern for suppliers regarding credit payments is the risk of specific customers failing to fulfill their payment obligations in full. Throughout the credit period, the seller also forgoes any potential interest earnings. To mitigate the risk of default, interest losses, and order cancellations, suppliers may prefer obtaining payment in advance. With this payment method, the supplier would require a complete payment before delivering the goods or services.

There are several potential benefits for a seller associated with receiving an advance payment. The probability of default is negligible, and cancellations of orders become unlikely, eliminating uncertainties. Prepayments also have the potential to generate interest income for the seller. However, if a vendor demands advance payment from a customer, the cost to the customer increases and the volume of sales decreases. On the contrary, accepting payment at a later facility enhances the sales amount, though it comes with potential downsides such as increased opportunity cost, default risk, or missed interest during the credit term, from the seller’s perspective. For sellers aiming to optimize profits, selecting the most advantageous payment mechanism, whether it’s advance, cash, or credit, poses an ongoing and persistent challenge. Following established principles in economics and marketing theory, it can be suggested that a drop in the cost of products or services should lead to an increase in demand. Therefore, pricing becomes a pivotal factor influencing both sales and profitability. Employing a strategy like price skimming, where a company initially sets a higher price and then lowers it as other products or services enter the market, can boost profits on new offerings. Customers may find it more economical to purchase a package deal from a small business rather than buying each item separately.

**Figure 1:**
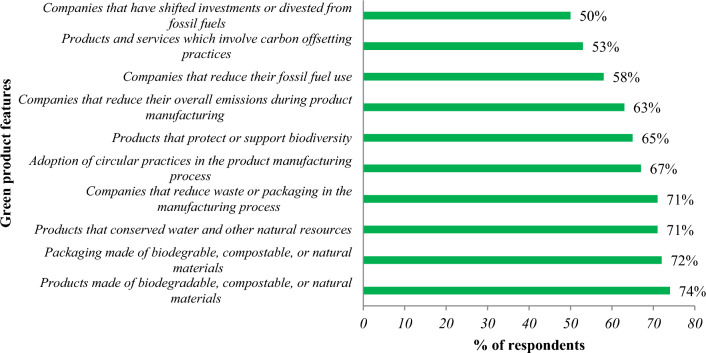
Green marketing promises that affected consumer purchasing in Australia in 2022.

Green products, also known as eco-friendly or sustainable products, are designed, promoted, and manufactured with the explicit objective of reducing their adverse environmental impacts throughout their entire life cycle. These products contribute to the advancement of sustainability and environmental stewardship by simultaneously decreasing resource use, waste generation, and pollution. The demand for sustainable and eco-friendly products is on the rise as customers become more conscious of the environmental effects of their purchase decisions. According to a separate Grand View Research report, the market for green products has experienced significant growth in recent years. The analysis estimates that the global market for green technology and sustainability was valued at USD 13.28 billion in 2021 and is projected to grow at a compound annual growth rate (CAGR) of 22.4% from 2022 to 2030^1^. Furthermore, a 2022 poll of Australian customers revealed that almost 74% of respondents were influenced to purchase a product based on the green promise that it was composed of natural, recyclable, or biodegradable ingredients (see Fig. 1). Additionally, nearly 72% of those polled stated that they were persuaded to buy a product by the assertion that the packaging was made of natural, reusable, or recyclable substances^2^. To maintain consumer demand and satisfaction, it is essential to ensure that green products are supplied and presented promptly while retaining their quality and environmental advantages. At this stage, this study addresses these research questions:

(i) How does the pricing of a green product impact consumer demand and purchasing habits?
(ii) What is the most effective pricing approach to strike a balance between market demand and profitability, considering the perceived value of the product’s environmental benefits?
(iii) What are the optimal inventory management practices for various payment methods, including advance payment, cash payment, and credit payment?
(iv) To what extent do consumer behavior and market competition influence the receptiveness of demand to changes in price, payment scheme duration, and the level of environmental sustainability?

These research questions examine the interactions between price, payment scheme duration, environmental sustainability, and customer demand for green products. This paper explores these issues and provides insights into profitable inventory and pricing strategies that optimize demand while promoting sustainable consumption.

### Literature review

Extensive scholarly literature on inventory models, encompassing credit payment mechanisms, is readily available. The study conducted by Chang et al.^3^ specifically delved into reviewing inventory models within the context of trade credits. A more contemporary review was subsequently presented by Seifert et al.^4^. This study will exclusively concentrate on issues directly correlated with the model under investigation.

In their study, Hwang and Shinn^5^ explored the simultaneous determination of pricing and lot size for an exponentially degrading good when the seller allows payment delays. To identify the optimal pricing and lot size for retailers utilizing a pay-later facility, Teng et al.^6^ researched an economic order quantity (EOQ) model, assuming that the product price exceeds the purchase cost. Teng et al.^7^ extended the investigation into the effects of trade credits on the economic production quantity (EPQ) model, building upon the EOQ model. Ho et al.^8^ introduced a demand-responsive integrated supplier-retailer supply chain system. The supplier also introduced a two-part trade-financing plan involving a cash reduction and a payment extension. A cash discount is granted to the store if the merchandise is paid off within a specified window of time; otherwise, the full purchase price is paid between the window of time and the final payment amount. To maximize overall profit, Ouyang et al.^9^ addressed the retailer’s order volume and the supplier’s manufacturing batch under an integrated EOQ model. Following a trade credit system outlined by Chang et al.^10^, buyers are granted a credit facility without interest if their purchase quantity meets or exceeds a predefined amount; otherwise, they are expected to pay for the items in full upon delivery. Thangam and Uthayakumar^11^ established optimal price and lot-sizing policies when the seller provides consumers with a partial trade credit policy and involves two warehouses for perishable items. According to this policy, half of the purchase price must be paid on credit, and the other half is due in full upon delivery. Jaggi et al.^12^ developed ideal ordering and pricing alternatives for merchants dealing with perishable goods in a two-warehouse system with fully backordered shortages. Investigating products that don't degrade quickly but have trade credit tied to order quantity, Chang et al.^13^ explored the best pricing and ordering methods. For goods with selling prices changing between non-deteriorating and deteriorating phases, Tsao et al.^14^ created a non-instantaneously degrading inventory model under pay-later facilities. Expanding on this model for products with maximum lifetimes, Tiwari et al.^15^ established it under allowable payment delays, allowing for partial backlogging and partial trade credits both upstream and downstream, as demonstrated by Mishra et al.^16^. In their study, Li and Teng^17^ explored pricing and lot-sizing rules for perishable goods, considering the impact of demand on selling price, reference price, product freshness, and displayed supplies. Subsequently, Panda et al.^18^ examined the effects of credit facilities within a two-warehouse inventory system. Das^19^ incorporated the simultaneous effects of reliability and trade credit on production management, while Khan et al.^20^ further enhanced quantity-based trade credit facilities within the context of limited capacity storage. It is crucial to note that the aforementioned research does not investigate the effects of credit facilities on the demand for sustainable green products. To address this gap, it is essential to explore an efficient inventory planning strategy for environmentally sustainable green products. This exploration should consider not only the level of environmental friendliness but also the credit length, as these factors have significant direct consequences on the consumption rates of these items.

An advanced payment method in inventory management involves acquiring goods through prepayment to suppliers or manufacturers. Maiti et al.^21^ investigated a model for inventory planning with advance payments, probabilistic lead-time, and price-dependent demand to optimize order quantity and pricing for maximizing expected profit. They employed a combination of the stochastic search genetic method and generalized reduced gradient methodology. Thangam^22^ developed optimal rates and quantities for perishable goods within a supply chain where the store receives upstream trade credit from the supplier and extends downstream trade credit to customers, implementing an advance-payment system. Taleizadeh^23^ examined a scenario in which a fuel provider required a petrol station to make a partial advance payment when placing an order and pay the entire amount in cash upon fuel delivery. Subsequently, Lashgari et al.^24^ developed an EOQ model incorporating partial upstream payments in advance and partial downstream financing payments, with or without shortages. Teng et al.^25^ delved into an EOQ model with advance payments specifically designed for decaying goods. Their assumption was that the rate of deterioration would visibly accelerate as the product approached its expiration date. Li et al.^26^ investigated joint inventory and pricing policies for degrading commodities covered by pay-later facilities. They posited that when a seller requests an advance-cash-credit payment from a customer, the demand is influenced by the combined effects of the selling price and product freshness. Taleizadeh et al.^27^ explored two distinct scenarios: complete advance payment with a shortfall and partial advance-partial pay later with a shortage, aiming to study the customer’s inventory strategy. Li et al.^28^ proposed an inventory model offering advance, cash, and credit as alternative payment terms to determine the collectively optimal ordering quantities. The model also identifies the ideal payment interval to maximize the retailer’s profit. Chang et al.^29^ investigated optimal pricing and lot-sizing methods for perishable goods, where the supplier mandates the producer to cover the entire transaction cost using a combination of cash, credit, and advance payments. Taleizadeh et al.^30^ presented an EOQ model with mixed sales incorporating multiple prepayments and partial credit payments. Subsequently, Khan et al.^31^ and Rahman et al.^32^ integrated discount facilities into a prepayment system while conducting inventory planning for deteriorating commodities. Manna et al.^33^ explored the prepayment mechanism in inventory planning for perishable goods, considering interval uncertainty. In a recent study by Khan et al.^34–36^, the authors determined the optimal frequency of installments for prepayment to minimize opportunity costs. Feng et al.^37^ discussed the timing of advanced payments for suppliers, considering the potential negative impact on demand. However, the mentioned research falls short in examining the prepayment mechanism related to sustainable green products. It is crucial to incorporate the impact of prepayment duration on demand when formulating an inventory model for sustainable green products, as it directly influences demand.

Understanding the demand for any item plays a pivotal role in devising effective inventory management strategies to sustain organizational operations. Jaggi et al.^38^ developed a credit-linked demand inventory model to address this significance. According to Feng et al.^39^, demand is shaped by factors such as the item’s selling price, displayed stock, and expiration date. Li et al.^40^ explored an inventory challenge with a demand-driven advance payment mechanism. Khan et al.^41^ conducted a study on an inventory model considering the impact of selling price on demand for perishable commodities with expiration dates. Rahman et al.^42^ took a parametric approach to address the issue in a demand-dependent inventory model characterized by interval-valued differential equations. According to Khan et al.^43^, a theoretical framework for inventory management suggests that the demand for deteriorating commodities is influenced by factors like selling price and advertisements, particularly in cases involving advance payment. Mishra et al.^44^ detailed the pricing and inventory decisions for deteriorating commodities under credit facilities, considering factors like selling price and stock amount affecting demand. Li et al.^45^ delved into the pricing, credit length, and order quantity dynamics for perishable commodities. Their study considered variables such as point-of-sale price, expiration date, and credit period influencing demand. As consumer preferences increasingly prioritize environmentally friendly and sustainable options, products aligned with these values tend to experience heightened demand. Saha et al.^46^ explored a model of a green supply chain incorporating both price-sensitive and green-sensitive demand. In a related study, Zand et al.^47^ examined an alternative supply chain framework incorporating both the greening level and the sensitivity of demand to price fluctuations. Additionally, Sana^48^ investigated the phenomenon of price rivalry between environmentally friendly (green) items and non-green products within the context of corporate social responsibility. In a study by Paul et al.^49^, the examination focused on the impact of the greening level on purchasing and selling prices. The study also incorporated the demand rate, revealing it to be a linearly rising function of the green level. Recently, Ali et al.^50^ explored the effects of products’ green levels on demand as a non-linear increasing function within a production inventory system. It is noteworthy that all the aforementioned research has treated demand as a variable dependent on either selling price, green level, or both. However, in the inventory modeling process for sustainable green products, none of them took into account the effects of credit length or prepayment duration on demand. The current work aims to effectively address this research gap.

‘Soft computing’ is a combination of computer techniques utilized to address problems that prove challenging for conventional methodologies. These algorithms are particularly employed when dealing with data that is challenging to express, possesses a significant level of uncertainty or ambiguity, or exhibits both characteristics. Soft computing methods find extensive applications in various domains such as robotics, image processing, data mining, and decision support systems. They prove especially valuable in situations requiring more adaptable and flexible approaches, or when conventional techniques fall short.

Swarm intelligence-based algorithms, physics-based algorithms, and evolutionary algorithms constitute the three different categories of meta-heuristics. Evolutionary algorithms, based on the principles of natural selection, find applications in optimization and search problems, while swarm intelligence algorithms draw inspiration from the collective behavior of social insects. In this field, Genetic Algorithm (GA) stands out as the most popular algorithm, modeling the concepts of Darwinian evolution and originally proposed by Holland^51^. An extensive investigation into the engineering applications of GA was carried out by Goldberg^52^. Evolutionary Algorithms (EAs) typically iterate on a starting random solution for optimization. Each new population is formed by combining and modifying members from the previous generation, with the expectation that the best individuals will contribute to its enhancement. This iterative process results in each succeeding generation likely being superior to the one before it. If this process is repeated over a large number of generations, the starting random population undergoes improvement. Some notable EAs include Biogeography-Based Optimizer (BBO) (Simon^53^), Evolutionary Programming (EP) (Yao et al.^54^), Evolution Strategy (ES), and Differential Evolution (DE) (Storm and Price^55^).

The second important topic in meta-heuristics is based on physics, where optimization methods often mimic physical rules. Notable algorithms in this category include Gravitational Local Search (GLSA) (Webster and Bernhard^56^), Galaxy-based Sea Search (Alatas^57^), Curved Space Optimization (CSO) (Moghaddam et al.^58^), and Ray Optimization (RO) algorithm (Kaveh and Khayatazad^59^). Unlike EAs, these physics-based algorithms employ a diverse group of search agents that interact and move around the search space in accordance with physical laws. For instance, the forces of gravity, ray casting, electromagnetic force, inertia force, weights, and other forces govern this movement.

**Figure 2:**
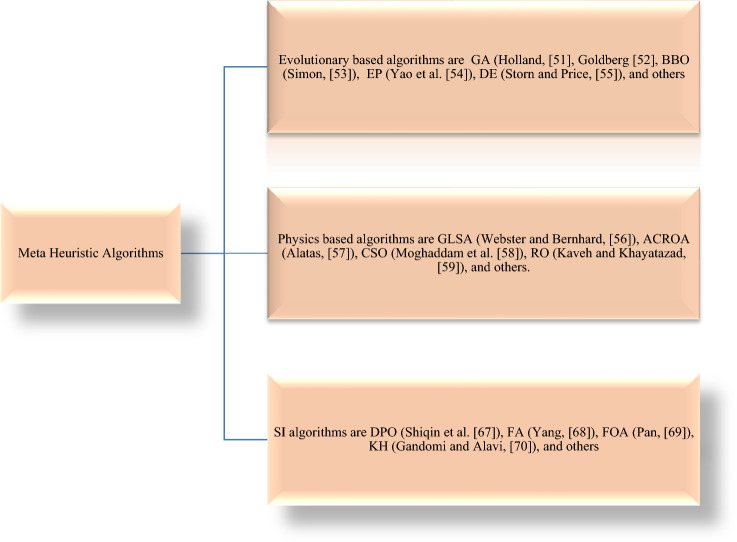
Different types of metaheuristic algorithms.

The third type of meta-heuristics comprises swarm intelligence (SI) techniques. These algorithms closely resemble the social behavior of naturally occurring living animals in swarms, herds, flocks, or schools. While they primarily employ a physics-based approach, these algorithms navigate by emulating the collective and social intelligence of living organisms. The most frequently applied SI method is Particle Swarm Optimization (PSO). Developed by Kennedy and Eberhart^60^, PSO algorithm is inspired by bird flocking. In PSO, numerous particles travel in line with the best particle and their own most advantageous locations to date. When moving a particle, both the best solution determined by the swarm and the particle’s own best solution are taken into account. Figure 2 provides a graphical representation of various SI algorithms, physics-based algorithms, and evolutionary algorithms.

### Contribution

The literature reviews above illustrate that studies have been conducted on prepayment mechanisms for both perishable and non-perishable commodities. However, there is a noticeable gap in research, with a lack of focus on effective inventory planning specifically for sustainable green products. Notably, the study by Feng et al.^37^ is the only one exploring the effects of payment time, specifically credit or prepayment periods, on the demand for conventional (non-green) products. However, their model is not suitable for sustainable green products, as the purchasing cost and consumption rate of these items are directly influenced by their green level. Additionally, to the best of the authors' knowledge, no study has investigated the consequences of prepayment duration on the demand rate for green products. Therefore, the current study aims to establish optimal inventory practices for companies dealing with sustainable green products. It considers the impacts of payment scheme periods, selling price, and green level on the consumption rate of these items. The following enumeration outlines the notable contributions of the present investigation:

- For the first time, this study presents a comparative analysis of the profitability of a seller engaged in the trade of sustainable green products, considering various payment options such as advance payment, cash payment, and credit payment.
- Identify the payment option that maximizes the seller’s profit, considering the nonlinear impact of payment duration, price, and green level on demand.
- The optimal pricing strategy is attained by effectively balancing market demand and profitability, all the while taking the perceived value of the product’s environmental benefits into account.
- This study investigates how default risk impacts the seller's optimal selling price and overall profit when offering downstream credit payments for sustainable green commodities.
- Examine the effect of a seller offering a price reduction for sustainable green commodities while requesting advance payment on the overall profitability.
- Due to the significant non-linearity observed in profit functions, optimization problems are addressed by utilizing TLBOA, which is widely recognized as a popular metaheuristic algorithm. Subsequently, the outcomes obtained from TLBOA are compared to those achieved via the use of GWOA and WOA.

### Orientation of the paper

The subsequent sections of this investigation are structured as follows: Section "Notation and assumptions" of the document presents the essential notation and assumptions required to effectively solve the problem. The formulation of the model for the advance payment, cash payment, and credit payment choices is presented in Section "Model formulation". Section "Solution methodology" of the document presents a concise overview of TLBOA, which is employed as a solution approach for the formulated maximization problems. In Section “Numerical illustration", several numerical examples are presented to illustrate the defined models, while Section "Sensitivity analyses" includes sensitivity assessments. To effectively operate a firm with the goal of generating profit, Sect. "Managerial insights" emphasizes the need for various management insights. The study’s findings are presented and summarized in Sect. "Conclusions".

## Notation and assumptions

The following notation and assumptions are used to develop the proposed model.

### Notation

**Table:** 

Symbol	Description
$$D_{1} \left( {L,p,g} \right)$$	Demand rate of the items
$$p$$	Selling price (in $) (a decision variable)
$$L$$	Payment period (in year) (a decision variable)
$$T$$	Business cycle length (in year) (a decision variable)
$$g$$	Green level of the products (a decision variable)
$$\xi$$	Index of green level in procuring cost
$$C_{p}$$	Purchase cost per unit (in $/unit)
$$C_{1} ,C_{2}$$	Purchasing cost parameters
$$C_{h}$$	Holding cost ($/unit item/year)
$$C_{o}$$	Ordering cost (in $/cycle)
$$K$$	Demand parameter
$$a$$	Payment period index in demand
$$\alpha$$	Payment period controlling scale parameter in demand
$$b$$	Index of the unit price in demand
$$\lambda$$	Selling price controlling scale parameter in demand
$$c$$	Index of green level in demand
$$\gamma$$	Green level controlling scale parameter in demand
$$r$$	Interest rate for earn /loss in advance payment and delay-in-payment risk
$$\, AP_{i} \left( {L,p,g,T} \right)$$	Average profit function under payment policy index $$i$$; $$i = 1$$ for Case-I: advance payment, $$i = 2$$ for Case-II: cash payment, and $$i = 3$$ for Case-III: credit payment (in $)

### Assumptions

The assumptions that have been taken into consideration in order to construct this proposed inventory model for sustainable green items are as follows:

(i) The inventory model is applicable to a seller who manages a single green item within an indefinite planning horizon.
(ii) There is no observed decline in quality or degradation during the duration of the stock period for the green item under consideration.
(iii) The purchasing cost of the products is regarded as an increasing function of the level of greenness of the items and mathematically, it is stated as: $C_{p} \left( g \right) = C_{1} + C_{2} g^{\xi }$, where $C_{1} ,\,\,C_{2} ,\,\,\xi > 0$.
(iv) Three different payment options, including advance payment, cash payment, and credit payment, are adopted in this study. In the case of advance payment, a discount is offered on the selling price of the item, and in the case of credit payment, a risk factor is considered in the recovery of sales revenue.
(v) The demand patterns under the three different payment schemes, viz., pay in advance (i.e., $L < 0$), cash payment (i.e., $L = 0$) and pay in later facility scheme (i.e., $L > 0$) are considered different. The additive influence of payment time, selling price, and green level on the consumption structure in power forms is being investigated. The mathematical expression of the demand rate is expressed as follows:
  $$D\left( {L,p,g} \right) = \left\{ {\begin{array}{*{20}c} {K + \alpha L^{a} - \lambda \left\{ {\left( {1 + d_{1} L} \right)p} \right\}^{b} + \gamma g^{c} ,\,\,{\text{when}}\,\,L < 0} \\ {K - \lambda p^{b} + \gamma g^{c} ,\,\,\,\,\,\,\,\,\,\,\,\,\,\,\,\,\,\,\,\,\,\,\,\,\,\,\,\,\,\,\,\,\,\,\,\,\,\,\,\,\,\,{\text{when}}\,\,L = 0} \\ {K + \alpha L^{a} - \lambda p^{b} + \gamma g^{c} ,\,\,\,\,\,\,\,\,\,\,\,\,\,\,\,\,\,\,\,\,\,\,\,\,\,\,\,{\text{when}}\,\,L > 0} \\ \end{array} } \right.,$$

where $\,K,\,\,\alpha ,\,\,\lambda ,\,\,d_{1} ,\,\,a,\,\,b,\,\,c\, > 0$. A longer prepayment duration is associated with a decrease in sales volume and an increase in interest earned for the seller (Khan et al.^61,62^). In order to mitigate the issue of low sales volume, it is common practice for a seller to provide clients with a price reduction (represented as $\left( {1 + d_{1} L} \right)p$) while requesting a prepayment (denoted as $L < 0$).

(vi) For a longer credit period, the default risk for the seller is also higher. Thus, in the case of credit payment, the default risk rate for any credit period $L > 0$ is considered as follows as $\left( {1 - e^{{ - d_{2} L}} } \right)$, where $d_{2} > 0$. Note that if $L = 0$, then the default risk rate becomes zero; on the other hand, if $L \to \infty$, then the default risk rate becomes 100%.
(vii) Shortages are not allowed in this proposed system.
(viii) A simple interest rate is considered on money that is either earned or lost.

## Model formulation

In the proposed model, the profit of the seller is formulated under three different payment categories. In an advance payment policy, the seller attracts customers by giving a discount on the selling price $p$. The discount depends on the advance payment period $L < 0$. For the delay-in-payment policy, there is a risk factor in earning sales revenue. Because of this risk factor, the seller does not allow any discounts on the product. For the cash payment scheme, the seller does not provide any discount on the selling price and the default risk factor rate is zero. Furthermore, the presence of eco-friendly items serves as a compelling factor that entices buyers to engage in increased purchasing behavior. Hence, the market is positively influenced by the demand that is dependent upon the green level $g$ of the product.

### Case-I: advance payment model $\left( {L < 0} \right)$

In various organizations, especially within the manufacturing and distribution sectors, a commonly observed practice involves offering an advance payment option accompanied by a discount. This approach encourages customers to make full advance payments, providing enterprises with expedited access to funds for operational expenses. Customers are motivated to make timely payments by receiving a deduction on the overall cost. This strategy benefits organizations by reducing the likelihood of bad debt, improving cash flow, and cutting administrative expenses associated with managing accounts receivable. Additionally, businesses can use the initial cash flow for operational needs, such as acquiring new machinery or expanding inventory. Customers can effectively manage their cash flow and lower buying expenses by taking advantage of the early payment discount. However, some consumers may face challenges in making an advance payment due to their own financial constraints or organizational protocols.

In this case, if $L < 0$, the advance payment mechanism occurs in the system. The governing differential equation for the inventory is as follows:

1 $$\frac{dI\left( t \right)}{{dt}} = - \left\{ {K + \alpha L^{a} - \lambda \left\{ {\left( {1 + d_{1} L} \right)p} \right\}^{b} + \gamma g^{c} } \right\},\,\,\,\,0 \le t \le T,$$

where $K,\,\,\alpha ,\,\,\lambda ,\,\,\gamma ,\,\,d_{1} ,\,\,a,\,\,b$, and $c$ are positive constants.

Solving Eq. (1), one can write

2 $$I\left( t \right) = \left\{ {K + \alpha L^{a} - \lambda \left\{ {\left( {1 + d_{1} L} \right)p} \right\}^{b} + \gamma g^{c} } \right\}\left( {T - t} \right),\,\,\,\,0 \le t \le T$$

Now, the items’ holding cost (*HC*) is given by

$$\begin{aligned} HC & = C_{h} \int\limits_{0}^{T} {I(t)dt} \\ & = C_{h} \left\{ {K + \alpha L^{a} - \lambda \left\{ {\left( {1 + d_{1} L} \right)p} \right\}^{b} + \gamma g^{c} } \right\}\frac{{T^{2} }}{2} \\ \end{aligned}$$

The sales revenue (*SR*) within each cycle is given by

$$SR = \left( {1 + d_{1} L} \right)p\left\{ {K + \alpha L^{a} - \lambda \left\{ {\left( {1 + d_{1} L} \right)p} \right\}^{b} + \gamma g^{c} } \right\}T$$

The purchase cost (*PC*) of the seller for the entire period is

$$\begin{aligned} PC & = C_{p} \left\{ {K + \alpha L^{a} - \lambda \left\{ {\left( {1 + d_{1} L} \right)p} \right\}^{b} + \gamma g^{c} } \right\}T \\ & = \left( {C_{1} + C_{2} g^{\xi } } \right)\left\{ {K + \alpha L^{a} - \lambda \left\{ {\left( {1 + d_{1} L} \right)p} \right\}^{b} + \gamma g^{c} } \right\}T \\ \end{aligned}$$

The seller receives revenue from 0 at the starting time $t = 0$ to $\left( {1 + d_{1} L} \right)p\left\{ {K + \alpha L^{a} - \lambda \left\{ {\left( {1 + d_{1} L} \right)p} \right\}^{b} + \gamma g^{c} } \right\}T$ at time $t = T$, and hence, the average revenue is $\frac{1}{2}\left( {1 + d_{1} L} \right)p\left\{ {K + \alpha L^{a} - \lambda \left\{ {\left( {1 + d_{1} L} \right)p} \right\}^{b} + \gamma g^{c} } \right\}T$. As a result, the interest earned by the seller from the average revenue is obtained by multiplying it with the interest rate $r$ and prepayment duration, that is, $- L$. Thus, the interest earned (*IE*) by the seller is given by

$$IE = - \frac{1}{2}rL\left( {1 + d_{1} L} \right)p\left\{ {K + \alpha L^{a} - \lambda \left\{ {\left( {1 + d_{1} L} \right)p} \right\}^{b} + \gamma g^{c} } \right\}T$$.

Since the ordering cost is $C_{o}$, the average profit function of the seller for the entire cycle is given by

3 $$\begin{gathered} AP_{1} \left( {L,p,g,T} \right) = \left( {1 - \frac{1}{2}rL} \right)\left( {1 + d_{1} L} \right)p\left\{ {K + \alpha L^{a} - \lambda \left\{ {\left( {1 + d_{1} L} \right)p} \right\}^{b} + \gamma g^{c} } \right\} \hfill \\ - C_{h} \left\{ {K + \alpha L^{a} - \lambda \left\{ {\left( {1 + d_{1} L} \right)p} \right\}^{b} + \gamma g^{c} } \right\}\frac{T}{2} - \left( {C_{1} + C_{2} g^{\xi } } \right)\left\{ {K + \alpha L^{a} - \lambda \left\{ {\left( {1 + d_{1} L} \right)p} \right\}^{b} + \gamma g^{c} } \right\} - \frac{{C_{o} }}{T} \hfill \\ \end{gathered}$$

Now, the corresponding optimization problem is stated as follows:

$$Maximize \, AP_{1} \left( {L,p,g,T} \right)$$

4 $${\text{Subject}}\,\,\,{\text{to}}\,\,0 < T,\,\,L > 0,\,\,p \ge 0,\,\,{\text{and}}\,\,g \ge 0.$$

### Case-II: cash payment model $\left( {L = 0} \right)$

In most business transactions, the conventional practice is to settle payment either upon the receipt of goods or after verifying their successful delivery. This strategy is grounded in the fundamental principles of trust and dependability, ensuring that buyers receive the expected products or services before releasing payment to the vendor. Such an approach not only fosters trust and diminishes the likelihood of conflicts but also promotes a seamless exchange of products and services, thereby enhancing the efficiency and security of the marketplace.

In this case, $L = 0$, i.e. no advance payment or delay in payment facilities are available. So, customers need to buy products with a cash payment. In this situation, the governing differential equation for the stock amount is as follows:

5 $$\frac{dI\left( t \right)}{{dt}} = - (K - \lambda p^{b} + \gamma g^{c} ),\,\,0 \le t \le T,$$

where $K,\,\,\lambda ,\,\,\gamma ,\,\,a,\,\,b$, and $c$ are positive constants.

Solving Eq. (5), one can write

6 $$I\left( t \right) = (K - \lambda p^{b} + \gamma g^{c} )\left( {T - t} \right),\,\,\,0 \le t \le T.$$

Now, the holding cost (*HC*) of the seller for the entire cycle is given by $HC = C_{h} \int\limits_{0}^{T} {I(t)dt} = C_{h} (K - \lambda p^{b} + \gamma g^{c} )\frac{{T^{2} }}{2}$.

The sales revenue (*SR*) earned by the seller during a complete cycle is given by

$$SR = p(K - \lambda p^{b} + \gamma g^{c} )T$$

In addition, the purchase cost (*PC*) of the seller for the entire period is given by

$$\begin{aligned} PC & = C_{p} (K - \lambda p^{b} + \gamma g^{c} )T \\ & = \left( {C_{1} + C_{2} g^{\xi } } \right)(K - \lambda p^{b} + \gamma g^{c} )T \\ \end{aligned}$$

Furthermore, the fixed ordering cost of the seller for a single cycle is $C_{o}$. Therefore, the average profit function of the seller for the entire cycle is given by

7 $$AP_{2} \left( {p,g,T} \right) = p(K - \lambda p^{b} + \gamma g^{c} ) - C_{h} (K - \lambda p^{b} + \gamma g^{c} )\frac{T}{2} - \left( {C_{1} + C_{2} g^{\xi } } \right)(K - \lambda p^{b} + \gamma g^{c} ) - \frac{{C_{o} }}{T}$$

Now, the corresponding optimization problem is stated as follows:

$$Maximize \, AP_{2} \left( {p,g,T} \right)$$

8 $${\text{Subject}}\,\,{\text{to}}\,\,0 < T,\,\,p \ge 0\,\,{\text{and}}\,\,g \ge 0.$$

### Case-III: credit payment model $\left( {L > 0} \right)$

In the current highly competitive corporate environment, a significant portion of transactions often take place through credit payment systems or deferred payment schemes, commonly known as ‘buy now, pay later’ (Akhtar et al.^63^). These payment options offer customers enhanced flexibility, enabling them to make purchases without the necessity of an immediate cash outlay. The popularity of credit cards, installment plans, and delayed payment services has grown, capturing customer interest for their convenient and accessible nature. This trend not only aligns with evolving consumer preferences but also empowers businesses to expand their client base and boost sales, underscoring the dynamic nature of contemporary commerce.

In this case, $L > 0$, i.e. a delay in payment facility is available. So, customers buy products with a credit payment facility. Thus, the governing differential equation of the inventory is as follows:

9 $$\frac{dI(t)}{{dt}} = - (K + \alpha L^{a} - \lambda p^{b} + \gamma g^{c} ),\,\,0 \le t \le T,$$

where $K,\,\,\alpha ,\,\,\lambda ,\,\,\gamma ,\,\,a,\,\,b$, and $c$ are positive constants.

By solving Eq. (9), one finds

10 $$I(t) = (K + \alpha L^{a} - \lambda p^{b} + \gamma g^{c} )\left( {T - t} \right),\,\,0 \le t \le T.$$

The holding cost (*HC*) of the seller for the entire cycle is given by

$$\begin{aligned} HC & = C_{h} \int\limits_{0}^{T} {I(t)dt} \\ & = C_{h} (K + \alpha L^{a} - \lambda p^{b} + \gamma g^{c} )\frac{{T^{2} }}{2} \\ \end{aligned}$$

Since the seller allows a credit period $L > 0$, there is a default risk with the rate $\left( {1 - e^{{ - d_{2} L}} } \right)$. Due to this default risk, the seller’s received sales revenue from each cycle is the revenue from sales multiplied by $e^{{ - d_{2} L}}$. Thus, the sales revenue (*SR*) earned by the seller due to the effect of risk factor is computed as follows:

$$SR = p(K + \alpha L^{a} - \lambda p^{b} + \gamma g^{c} )Te^{{ - d_{2} L}} .$$

Furthermore, as the seller allows a credit period $L > 0$, there is an opportunity loss to earn interest from the sales revenue. Therefore, the interest loss (*IL*) from each cycle is the average revenue, that is, $\frac{1}{2}p(K + \alpha L^{a} - \lambda p^{b} + \gamma g^{c} )T$ times the interest rate $r$ multiplied by the credit duration $L > 0$, which is computed as follows:

$$IL = \frac{1}{2}rLp(K + \alpha L^{a} - \lambda p^{b} + \gamma g^{c} )T.$$

Moreover, the purchase cost (*PC*) of the seller for the entire period is given by

$$PC = C_{p} (K + \alpha L^{a} - \lambda p^{b} + \gamma g^{c} )T = \left( {C_{1} + C_{2} g^{\xi } } \right)(K + \alpha L^{a} - \lambda p^{b} + \gamma g^{c} )T.$$

As the fixed ordering cost of the seller for a single cycle is $C_{o}$, the average profit function of the seller for the entire cycle is given by

11 $$\begin{gathered} AP_{3} \left( {L,p,g,T} \right) = p(K + \alpha L^{a} - \lambda p^{b} + \gamma g^{c} )e^{{ - d_{2} L}} - \frac{1}{2}rLp(K + \alpha L^{a} - \lambda p^{b} + \gamma g^{c} ) \hfill \\ \,\,\,\,\,\,\,\,\,\,\,\,\,\,\,\,\,\,\,\,\,\,\,\,\,\,\,\,\,\,\,\,\,\,\,\,\,\,\, - C_{h} (K + \alpha L^{a} - \lambda p^{b} + \gamma g^{c} )\frac{T}{2} - \left( {C_{1} + C_{2} g^{\xi } } \right)(K + \alpha L^{a} - \lambda p^{b} + \gamma g^{c} ) - \frac{{C_{o} }}{T}. \hfill \\ \end{gathered}$$

Therefore, the corresponding maximization problem is

$$Maximize \, AP_{3} \left( {L,p,g,T} \right)$$

12 $${\text{Subject }}\,\,{\text{to}}\,\,{\text{0 < T, }}\,\,L > 0,\,\,p \ge 0\,\,\,{\text{and}}\,\,{\text{g}} \ge 0.$$

## Solution methodology

First, the optimality of all decision variables in the formulated nonlinear maximization problems (4), (8), and (12) is investigated. For the profit function $AP_{1} \left( {L,p,g,T} \right)$ of the seller under an advance payment scheme, all the first-order partial derivatives with respect to $L$, $p$, $g$, and $T$ are found as

13 $$\begin{gathered} \frac{{\partial AP_{1} \left( {L,p,g,T} \right)}}{\partial L} = \left( {d_{1} - \frac{1}{2}r - rd_{1} L} \right)p\left\{ {K + \alpha L^{a} - \lambda \left\{ {\left( {1 + d_{1} L} \right)p} \right\}^{b} + \gamma g^{c} } \right\} \hfill \\ \,\,\,\,\,\,\,\,\,\,\,\,\,\,\,\,\,\,\,\,\,\,\,\,\,\,\,\,\,\,\,\,\,\,\,\,\,\, + \left\{ {\left( {1 - \frac{1}{2}rL} \right)\left( {1 + d_{1} L} \right)p - \left( {\frac{{C_{h} T}}{2} + C_{1} + C_{2} g^{\xi } } \right)} \right\}\left\{ {\alpha aL^{a - 1} - \lambda bd_{1} p\left\{ {\left( {1 + d_{1} L} \right)p} \right\}^{b - 1} } \right\}, \hfill \\ \end{gathered}$$

14 $$\begin{gathered} \frac{{\partial AP_{1} \left( {L,p,g,T} \right)}}{\partial p} = \left( {1 - \frac{1}{2}rL} \right)\left( {1 + d_{1} L} \right)\left\{ {K + \alpha L^{a} - \lambda \left\{ {\left( {1 + d_{1} L} \right)p} \right\}^{b} + \gamma g^{c} } \right\} \hfill \\ \,\,\,\,\,\,\,\,\,\,\,\,\,\,\,\,\,\,\,\,\,\,\,\,\,\,\,\,\,\,\,\,\,\,\,\,\,\,\,\,\,\,\,\,\,\, - \left( {1 - \frac{1}{2}rL} \right)\left( {1 + d_{1} L} \right)^{b + 1} \lambda bp^{b} + \left( {C_{h} \frac{T}{2} + C_{1} + C_{2} g^{\xi } } \right)\lambda b\left( {1 + d_{1} L} \right)^{b} p^{b - 1} , \hfill \\ \end{gathered}$$

15 $$\begin{gathered} \frac{{\partial AP_{1} \left( {L,p,g,T} \right)}}{\partial g} = \left( {1 - \frac{1}{2}rL} \right)\left( {1 + d_{1} L} \right)p\gamma cg^{c - 1} - c\gamma g^{c - 1} \left( {C_{h} \frac{T}{2} + C_{1} + C_{2} g^{\xi } } \right) \hfill \\ \,\,\,\,\,\,\,\,\,\,\,\,\,\,\,\,\,\,\,\,\,\,\,\,\,\,\,\,\,\,\,\,\,\,\,\,\,\,\,\,\,\,\,\,\, - \left( {C_{2} \xi g^{\xi - 1} } \right)\left\{ {K + \alpha L^{a} - \lambda \left\{ {\left( {1 + d_{1} L} \right)p} \right\}^{b} + \gamma g^{c} } \right\}, \hfill \\ \end{gathered}$$

16 $$\frac{{\partial AP_{1} \left( {L,p,g,T} \right)}}{\partial T} = - \frac{{C_{h} }}{2}\left\{ {K + \alpha L^{a} - \lambda \left\{ {\left( {1 + d_{1} L} \right)p} \right\}^{b} + \gamma g^{c} } \right\} + \frac{{C_{o} }}{{T^{2} }}.$$

Solving the necessary conditions $\frac{{\partial AP_{1} \left( {L,p,g,T} \right)}}{\partial L} = 0$, $\frac{{\partial AP_{1} \left( {L,p,g,T} \right)}}{\partial p} = 0$, $\frac{{\partial AP_{1} \left( {L,p,g,T} \right)}}{\partial g} = 0$, and $\frac{{\partial AP_{1} \left( {L,p,g,T} \right)}}{\partial T} = 0$ simultaneously, one can find the optimal solutions for the seller. However, for all $a,\,b,\,c$, and $\xi$, the closed-form solutions from these necessary conditions are not possible as the indices $a,\,b,\,c$, and $\xi$ maintain the inequalities $a,\,b,\,c,\xi > 0$. Furthermore, the Hessian matrix of $AP_{1} \left( {L,p,g,T} \right)$ with respect to $L$, $p$, $g$, and $T$ is

$$H^{(1)} = \left[ {\begin{array}{*{20}c} {\frac{{\partial^{2} AP_{1} }}{{\partial L^{2} }}} & {\frac{{\partial^{2} AP_{1} }}{\partial L\partial p}} & {\frac{{\partial^{2} AP_{1} }}{\partial L\partial g}} & {\frac{{\partial^{2} AP_{1} }}{\partial L\partial T}} \\ {\frac{{\partial^{2} AP_{1} }}{\partial p\partial L}} & {\frac{{\partial^{2} AP_{1} }}{{\partial p^{2} }}} & {\frac{{\partial^{2} AP_{1} }}{\partial p\partial g}} & {\frac{{\partial^{2} AP_{1} }}{\partial p\partial T}} \\ {\frac{{\partial^{2} AP_{1} }}{\partial g\partial L}} & {\frac{{\partial^{2} AP_{1} }}{\partial g\partial p}} & {\frac{{\partial^{2} AP_{1} }}{{\partial g^{2} }}} & {\frac{{\partial^{2} AP_{1} }}{\partial g\partial T}} \\ {\frac{{\partial^{2} AP_{1} }}{\partial T\partial L}} & {\frac{{\partial^{2} AP_{1} }}{\partial T\partial p}} & {\frac{{\partial^{2} AP_{1} }}{\partial T\partial g}} & {\frac{{\partial^{2} AP_{1} }}{{\partial T^{2} }}} \\ \end{array} } \right]$$, 

where

$\frac{{\partial^{2} AP_{1} }}{{\partial L^{2} }} = X_{1} - X_{2}$, $\frac{{\partial^{2} AP_{1} }}{\partial L\partial p} = X_{3}$, $\frac{{\partial^{2} AP_{1} }}{\partial L\partial g} = X_{4}$, $\frac{{\partial^{2} AP_{1} }}{\partial L\partial T} = - X_{5}$, $\frac{{\partial^{2} AP_{1} }}{\partial p\partial L} = X_{3}$, $\frac{{\partial^{2} AP_{1} }}{{\partial p^{2} }} = X_{6}$, $\frac{{\partial^{2} AP_{1} }}{\partial p\partial g} = X_{7}$, $\frac{{\partial^{2} AP_{1} }}{\partial p\partial T} = X_{8}$, $\frac{{\partial^{2} AP_{1} }}{\partial g\partial L} = X_{4}$, $\frac{{\partial^{2} AP_{1} }}{\partial g\partial p} = X_{7}$, $\frac{{\partial^{2} AP_{1} }}{{\partial g^{2} }} = X_{9}$, $\frac{{\partial^{2} AP_{1} }}{\partial g\partial T} = - X_{10}$, $\frac{{\partial^{2} AP_{1} }}{\partial T\partial L} = - X_{5}$, $\frac{{\partial^{2} AP_{1} }}{\partial T\partial p} = X_{8}$ , $\frac{{\partial^{2} AP_{1} }}{\partial T\partial g} = - X_{10}$, $\frac{{\partial^{2} AP_{1} }}{{\partial T^{2} }} = - X_{11}$. In addition, the expressions of $X_{i}$ ($i = 1,2,...,11$) are supplied in supplementary material in Appendix A. Now, the principal minors of the Hessian matrix $H^{(1)}$ are as follows:

17 $$\left| {H_{11}^{(1)} } \right| = X_{1} - X_{2} < 0,\,\,{\text{if }}\,{\text{X}}_{{1}} \,{ < }\,{\text{X}}_{{2}} \,\,,$$

18 $$\left| {H_{22}^{(1)} } \right| = X_{6} (X_{1} - X_{2} ) - X_{3}^{2} > 0,\,\,{\text{if}}\,\,X_{1} X_{6} > X_{2} X_{6} + X_{3}^{2} ,$$

$$\left| {H_{33}^{(1)} } \right| = (X_{1} - X_{2} )(X_{6} X_{9} - X_{7}^{2} ) - X_{3} (X_{3} X_{9} - X_{4} X_{7} ) + X_{4} (X_{3} X_{7} - X_{4} X_{6} ) < 0$$,

19 $${\text{if}}\,X_{1} X_{6} X_{9} + 2X_{3} X_{4} X_{7} + X_{2} X_{7}^{2} < X_{2} X_{6} X_{9} + X_{1} X_{7}^{2} + X_{3}^{2} X_{9} + X_{4}^{2} X_{6}$$

and

$$\begin{gathered} \left| {H_{44}^{(1)} } \right| = (X_{1} - X_{2} )\left\{ { - X_{6} (X_{9} X_{11} + X_{10}^{2} ) - X_{7} ( - X_{7} X_{11} + X_{8} X_{10} ) - X_{8} (X_{7} X_{10} + X_{8} X_{9} )} \right\} \hfill \\ \,\,\,\,\,\,\,\,\,\,\,\,\,\,\,\,\,\, - X_{3} \left\{ { - X_{3} (X_{9} X_{11} + X_{10}^{2} ) + X_{7} (X_{4} X_{11} + X_{5} X_{10} ) + X_{8} ( - X_{4} X_{10} + X_{5} X_{9} )} \right\} \hfill \\ \,\,\,\,\,\,\,\,\,\,\,\,\,\,\,\,\,\, + X_{4} \left\{ {X_{3} ( - X_{7} X_{11} + X_{8} X_{10} ) + X_{6} (X_{4} X_{11} + X_{5} X_{10} ) + X_{8} (X_{4} X_{8} + X_{5} X_{7} )} \right\} \hfill \\ \,\,\,\,\,\,\,\,\,\,\,\,\,\,\,\,\,\, + X_{5} \left\{ { - X_{3} (X_{7} X_{10} + X_{8} X_{9} ) - X_{6} ( - X_{4} X_{10} + X_{5} X_{9} ) + X_{7} (X_{4} X_{8} + X_{5} X_{7} )} \right\} > 0, \hfill \\ \end{gathered}$$

if

20 $$\begin{gathered} (X_{2} X_{6} X_{9} X_{11} + 2X_{2} X_{7} X_{8} X_{10} + 2X_{3} X_{4} X_{8} X_{10} + 2X_{4} X_{5} X_{6} X_{10} + 2X_{4} X_{5} X_{7} X_{8} \hfill \\ + X_{1} X_{7}^{2} X_{11} + X_{2} X_{6} X_{10}^{2} + X_{2} X_{8}^{2} X_{9} + X_{3}^{2} X_{9} X_{11} + X_{4}^{2} X_{6} X_{11} + X_{3}^{2} X_{10}^{2} + X_{4}^{2} X_{8}^{2} + X_{5}^{2} X_{7}^{2} ) > \hfill \\ (X_{1} X_{6} X_{9} X_{11} + 2X_{1} X_{7} X_{8} X_{10} + 2X_{3} X_{4} X_{7} X_{11} + 2X_{3} X_{5} X_{7} X_{10} + 2X_{3} X_{5} X_{8} X_{9} \hfill \\ + X_{1} X_{6} X_{10}^{2} + X_{1} X_{8}^{2} X_{9} + X_{2} X_{7}^{2} X_{11} + X_{5}^{2} X_{6} X_{9} ). \hfill \\ \end{gathered}$$

Based on the aforementioned formulations, it can be inferred that the determination of the global optimal solutions for the presented problem is possible if the conditions (17), (18), (19), (20) are satisfied.

To investigate the optimality for the second profit function $AP_{2} \left( {p,g,T} \right)$, compute all the first-order partial derivatives with respect to $p$, $g$, and $T$ as follows:

21 $$\frac{{\partial AP_{2} \left( {p,g,T} \right)}}{\partial p} = K - \lambda (b + 1)p^{b} + \gamma g^{c} + \left( {\frac{1}{2}C_{h} T + C_{1} + C_{2} g^{\xi } } \right)\lambda bp^{b - 1} ,$$

22 $$\frac{{\partial AP_{2} \left( {p,g,T} \right)}}{\partial g} = \left( {p - \frac{1}{2}C_{h} T - C_{1} - C_{2} g^{\xi } } \right)\gamma cg^{c - 1} - \left( {C_{1} + C_{2} \xi g^{\xi - 1} } \right)(K - \lambda p^{b} + \gamma g^{c} ),$$

23 $$\frac{{\partial AP_{2} \left( {p,g,T} \right)}}{\partial T} = - \frac{1}{2}C_{h} (K - \lambda p^{b} + \gamma g^{c} ) + \frac{{C_{o} }}{{T^{2} }},$$

By solving the necessary conditions $\frac{{\partial AP_{2} \left( {p,g,T} \right)}}{\partial p} = 0$, $\frac{{\partial AP_{2} \left( {p,g,T} \right)}}{\partial g} = 0$, and $\frac{{\partial AP_{2} \left( {p,g,T} \right)}}{\partial T} = 0$ simultaneously, one can find the optimal solutions for the seller. However, for all $b,\,c$, and $\xi$, the closed-form solutions from these necessary conditions are not possible as the indices $b,\,c$, and $\xi$ maintain the inequalities $b,\,c,\xi > 0$. In addition, the Hessian matrix of $AP_{2} \left( {p,g,T} \right)$ with respect to $p$, $g$, and $T$ is

$$H^{(2)} = \left[ {\begin{array}{*{20}c} {\frac{{\partial^{2} AP_{2} }}{{\partial p^{2} }}} & {\frac{{\partial^{2} AP_{2} }}{\partial p\partial g}} & {\frac{{\partial^{2} AP_{2} }}{\partial p\partial T}} \\ {\frac{{\partial^{2} AP_{2} }}{\partial g\partial p}} & {\frac{{\partial^{2} AP_{2} }}{{\partial g^{2} }}} & {\frac{{\partial^{2} AP_{2} }}{\partial g\partial T}} \\ {\frac{{\partial^{2} AP_{2} }}{\partial T\partial p}} & {\frac{{\partial^{2} AP_{2} }}{\partial T\partial g}} & {\frac{{\partial^{2} AP_{2} }}{{\partial T^{2} }}} \\ \end{array} } \right]$$, 

where

$\frac{{\partial^{2} AP_{2} }}{{\partial p^{2} }} = Y_{1} - Y_{2}$, $\frac{{\partial^{2} AP_{2} }}{\partial p\partial g} = Y_{3}$, $\frac{{\partial^{2} AP_{2} }}{\partial p\partial T} = Y_{4}$, $\frac{{\partial^{2} AP_{2} }}{\partial g\partial p} = Y_{3}$, $\frac{{\partial^{2} AP_{2} }}{{\partial g^{2} }} = Y_{5}$, $\frac{{\partial^{2} AP_{2} }}{\partial g\partial T} = - Y_{6}$, $\frac{{\partial^{2} AP_{2} }}{\partial T\partial p} = Y_{4}$, $\frac{{\partial^{2} AP_{2} }}{\partial T\partial g} = - Y_{6}$, $\frac{{\partial^{2} AP_{2} }}{{\partial T^{2} }} = - Y_{7}$. Furthermore, the expressions of $Y_{i}$ ($i = 1,2,...,7$) are provided in supplementary material in Appendix B. The principal minors of the Hessian matrix $H^{(2)}$ are shown here.

24 $$\left| {H_{11}^{(2)} } \right| = Y_{1} - Y_{2} < 0,\,\,{\text{if}}\,Y_{1} < Y_{2} ,$$

25 $$\left| {H_{22}^{(2)} } \right| = Y_{5} (Y_{1} - Y_{2} ) - Y_{3}^{2} > 0,\,\,{\text{if}}\,\,Y_{1} Y_{5} > Y_{2} Y_{5} + Y_{3}^{2} .$$

and

$$\left| {H_{33}^{(2)} } \right| = (Y_{1} - Y_{2} )( - Y_{5} Y_{7} - Y_{6}^{2} ) - Y_{3} ( - Y_{3} Y_{7} + Y_{4} Y_{6} ) + Y_{4} ( - Y_{3} Y_{6} - Y_{4} Y_{5} ) < 0$$,

if

26 $$Y_{2} Y_{5} Y_{7} + Y_{2} Y_{6}^{2} + Y_{3}^{2} Y_{7} < Y_{1} Y_{5} Y_{7} + 2Y_{3} Y_{4} Y_{6} + Y_{1} Y_{6}^{2} + Y_{4}^{2} Y_{5} .$$

From the above formulations, it can be deduced that if the requirements (24), (25), (26) are met, it is feasible to determine the global optimal solutions for the second profit function $AP_{2} \left( {p,g,T} \right)$.

Finally, in order to examine the optimality of the third profit function $AP_{3} \left( {L,p,g,T} \right)$, it is necessary to calculate the first-order partial derivatives with respect to $L$, $p$, $g$, and $T$, as outlined below.

27 $$\begin{gathered} \frac{{\partial AP_{3} \left( {L,p,g,T} \right)}}{\partial L} = p\alpha aL^{a - 1} e^{{ - d_{2} L}} - d_{2} p(K + \alpha L^{a} - \lambda p^{b} + \gamma g^{c} )e^{{ - d_{2} L}} \hfill \\ \,\,\,\,\,\,\,\,\,\,\,\,\,\,\,\,\,\,\,\,\,\,\,\,\,\,\,\,\,\,\,\,\,\,\,\,\,\,\,\,\,\, - \frac{1}{2}rp(K + \alpha L^{a} - \lambda p^{b} + \gamma g^{c} ) - \frac{1}{2}rp\alpha aL^{a} - \left( {\frac{T}{2}C_{h} + C_{1} + C_{2} g^{\xi } } \right)\alpha aL^{a - 1} , \hfill \\ \end{gathered}$$

28 $$\frac{{\partial AP_{3} \left( {L,p,g,T} \right)}}{\partial p} = \{ K + \alpha L^{a} - \lambda (b + 1)p^{b} + \gamma g^{c} \} \left( {e^{{ - d_{2} L}} - \frac{1}{2}rL} \right) + \left( {\frac{T}{2}C_{h} + C_{1} + C_{2} g^{\xi } } \right)\lambda bp^{b - 1} ,$$

29 $$\begin{gathered} \frac{{\partial AP_{3} \left( {L,p,g,T} \right)}}{\partial g} = \left( {e^{{ - d_{2} L}} - \frac{1}{2}rL} \right)p\gamma cg^{c - 1} - \left( {\frac{T}{2}C_{h} + C_{1} + C_{2} g^{\xi } } \right)\gamma cg^{c - 1} \hfill \\ \,\,\,\,\,\,\,\,\,\,\,\,\,\,\,\,\,\,\,\,\,\,\,\,\,\,\,\,\,\,\,\,\,\,\,\,\,\,\,\,\,\,\,\,\,\,\,\,\,\,\,\,\,\, - C_{2} \xi g^{\xi - 1} (K + \alpha L^{a} - \lambda p^{b} + \gamma g^{c} ), \hfill \\ \end{gathered}$$

30 $$\frac{{\partial AP_{3} \left( {L,p,g,T} \right)}}{\partial T} = - \frac{1}{2}C_{h} (K + \alpha L^{a} - \lambda p^{b} + \gamma g^{c} ) + \frac{{C_{o} }}{{T^{2} }}.$$

The optimal solutions for the seller can be found by solving the necessary conditions $\frac{{\partial AP_{3} \left( {L,p,g,T} \right)}}{\partial L} = 0$, $\frac{{\partial AP_{3} \left( {L,p,g,T} \right)}}{\partial p} = 0$, $\frac{{\partial AP_{3} \left( {L,p,g,T} \right)}}{\partial g} = 0$, and $\frac{{\partial AP_{3} \left( {L,p,g,T} \right)}}{\partial T} = 0$ simultaneously. It is noteworthy that the closed-form solutions from these necessary conditions are not possible for all $a,\,b,\,c$, and $\xi$, as these indices maintain the inequalities $a,\,b,\,c,\xi > 0$. Additionally, the Hessian matrix of $AP_{3} \left( {L,p,g,T} \right)$ with respect to $L$, $p$, $g$, and $T$ is

$$H^{(3)} = \left[ {\begin{array}{*{20}c} {\frac{{\partial^{2} AP_{3} }}{{\partial L^{2} }}} & {\frac{{\partial^{2} AP_{3} }}{\partial L\partial p}} & {\frac{{\partial^{2} AP_{3} }}{\partial L\partial g}} & {\frac{{\partial^{2} AP_{3} }}{\partial L\partial T}} \\ {\frac{{\partial^{2} AP_{3} }}{\partial p\partial L}} & {\frac{{\partial^{2} AP_{3} }}{{\partial p^{2} }}} & {\frac{{\partial^{2} AP_{3} }}{\partial p\partial g}} & {\frac{{\partial^{2} AP_{3} }}{\partial p\partial T}} \\ {\frac{{\partial^{2} AP_{3} }}{\partial g\partial L}} & {\frac{{\partial^{2} AP_{3} }}{\partial g\partial p}} & {\frac{{\partial^{2} AP_{3} }}{{\partial g^{2} }}} & {\frac{{\partial^{2} AP_{3} }}{\partial g\partial T}} \\ {\frac{{\partial^{2} AP_{3} }}{\partial T\partial L}} & {\frac{{\partial^{2} AP_{3} }}{\partial T\partial p}} & {\frac{{\partial^{2} AP_{3} }}{\partial T\partial g}} & {\frac{{\partial^{2} AP_{3} }}{{\partial T^{2} }}} \\ \end{array} } \right]$$,

where

$\frac{{\partial^{2} AP_{3} }}{{\partial L^{2} }} = Z_{1} - Z_{2}$, $\frac{{\partial^{2} AP_{3} }}{\partial L\partial p} = Z_{3}$, $\frac{{\partial^{2} AP_{3} }}{\partial L\partial g} = - Z_{4}$, $\frac{{\partial^{2} AP_{3} }}{\partial L\partial T} = - Z_{5}$, $\frac{{\partial^{2} AP_{3} }}{\partial p\partial L} = Z_{3}$, $\frac{{\partial^{2} AP_{3} }}{{\partial p^{2} }} = Z_{6}$, $\frac{{\partial^{2} AP_{3} }}{\partial p\partial g} = Z_{7}$, $\frac{{\partial^{2} AP_{3} }}{\partial p\partial T} = Z_{8}$, $\frac{{\partial^{2} AP_{3} }}{\partial g\partial L} = - Z_{4}$, $\frac{{\partial^{2} AP_{3} }}{\partial g\partial p} = Z_{7}$, $\frac{{\partial^{2} AP_{3} }}{{\partial g^{2} }} = Z_{9}$, $\frac{{\partial^{2} AP_{3} }}{\partial g\partial T} = - Z_{10}$, $\frac{{\partial^{2} AP_{3} }}{\partial T\partial L} = - Z_{5}$, $\frac{{\partial^{2} AP_{3} }}{\partial T\partial p} = Z_{8}$, $\frac{{\partial^{2} AP_{3} }}{\partial T\partial g} = - Z_{10}$, $\frac{{\partial^{2} AP_{3} }}{{\partial T^{2} }} = - Z_{11}$. In addition, the expressions of $Z_{i}$ ($i = 1,2,...,11$) are supplied in supplementary material in Appendix C. Now, the principal minors of the Hessian matrix $H^{(1)}$ are as follows:

31 $$\left| {H_{11}^{(3)} } \right| = Z_{1} - Z_{2} < 0,\,\,{\text{if}}\,\,Z_{1} < Z_{2} ,$$

32 $$\left| {H_{22}^{(3)} } \right| = Z_{6} (Z_{1} - Z_{2} ) - Z_{3}^{2} > 0,\,\,{\text{if}}\,\,Z_{1} Z_{6} > Z_{2} Z_{6} + Z_{3}^{2} ,$$

$$\left| {H_{33}^{(3)} } \right| = (Z_{1} - Z_{2} )(Z_{6} Z_{9} - Z_{7}^{2} ) - Z_{3} (Z_{3} Z_{9} + Z_{4} Z_{7} ) - Z_{4} (Z_{3} Z_{7} + Z_{4} Z_{6} ) < 0,$$

if

33 $$Z_{1} Z_{6} Z_{9} + Z_{2} Z_{7}^{2} < 2Z_{3} Z_{4} Z_{7} + Z_{2} Z_{6} Z_{9} + Z_{1} Z_{7}^{2} + Z_{3}^{2} Z_{9} + Z_{4}^{2} Z_{6} ,$$

and

$$\begin{gathered} \left| {H_{44}^{(3)} } \right| = (Z_{1} - Z_{2} )\left\{ { - Z_{6} (Z_{9} Z_{11} + Z_{10}^{2} ) - Z_{7} ( - Z_{7} Z_{11} + Z_{8} Z_{10} ) - Z_{8} (Z_{7} Z_{10} + Z_{8} Z_{9} )} \right\} \hfill \\ \,\,\,\,\,\,\,\,\,\,\,\,\,\,\,\,\,\, - Z_{3} \left\{ { - Z_{3} (Z_{9} Z_{11} + Z_{10}^{2} ) + Z_{7} ( - Z_{4} Z_{11} + Z_{5} Z_{10} ) + Z_{8} (Z_{4} Z_{10} + Z_{5} Z_{9} )} \right\} \hfill \\ \,\,\,\,\,\,\,\,\,\,\,\,\,\,\,\,\,\, - Z_{4} \left\{ {Z_{3} ( - Z_{7} Z_{11} + Z_{8} Z_{10} ) + Z_{6} ( - Z_{4} Z_{11} + Z_{5} Z_{10} ) + Z_{8} ( - Z_{4} Z_{8} + Z_{5} Z_{7} )} \right\} \hfill \\ \,\,\,\,\,\,\,\,\,\,\,\,\,\,\,\,\,\, + Z_{5} \left\{ { - Z_{3} (Z_{7} Z_{10} + Z_{8} Z_{9} ) - Z_{6} (Z_{4} Z_{10} + Z_{5} Z_{9} ) + Z_{7} ( - Z_{4} Z_{8} + Z_{5} Z_{7} )} \right\} > 0, \hfill \\ \end{gathered}$$

if

34 $$\begin{gathered} (Z_{2} Z_{6} Z_{9} Z_{11} + 2Z_{2} Z_{7} Z_{8} Z_{10} + 2Z_{3} Z_{4} Z_{7} Z_{11} + Z_{1} Z_{7}^{2} Z_{11} + Z_{2} Z_{6} Z_{10}^{2} + Z_{2} Z_{8}^{2} Z_{9} + Z_{3}^{2} Z_{9} Z_{11} \hfill \\ + Z_{4}^{2} Z_{6} X_{11} + Z_{3}^{2} Z_{10}^{2} + Z_{4}^{2} X_{8}^{2} + Z_{5}^{2} Z_{7}^{2} ) > (Z_{1} Z_{6} Z_{9} Z_{11} + 2Z_{1} Z_{7} Z_{8} Z_{10} + 2Z_{3} Z_{5} Z_{7} Z_{10} + 2Z_{3} Z_{5} Z_{8} Z_{9} \hfill \\ + 2Z_{3} Z_{4} Z_{8} Z_{10} + 2Z_{4} Z_{5} Z_{6} Z_{10} + 2Z_{4} Z_{5} Z_{7} Z_{8} + Z_{1} Z_{6} Z_{10}^{2} + Z_{1} Z_{8}^{2} Z_{9} + Z_{2} Z_{7}^{2} Z_{11} + Z_{5}^{2} Z_{6} Z_{9} ). \hfill \\ \end{gathered}$$

From the aforementioned formulations, it is possible to find the global optimal solutions for the third profit function $AP_{3} \left( {L,p,g,T} \right)$, provided that conditions (31) through (34) are satisfied. It is important to acknowledge that the process of analytically verifying all the necessary requirements is not only arduous but also time-consuming. Therefore, the aforementioned problems (4), (8), and (12) are solved and thereafter evaluated by the utilization of several efficient meta-heuristic methods.

The problems outlined in Eqs. (4), (8), and (12) pose a challenge as they do not allow for the derivation of a closed-form solution for the decision variables. This difficulty arises from the escalating nonlinearity present in the objective functions $\, AP_{i} \left( {L,p,g,T} \right),\,\,i = 1,2,3$. In addition, when these problems are solved with the help of optimization tools like MATHEMATICA, LINGO, or Python for a given set of data, shortcomings like "the solution fails to reach convergence within the maximum default value of iteration" appear, or sometimes they provide immature solutions for specific optimisation problems. As the greatest number of iterations can be altered to suit the requirements of obtaining the best (near-optimal) solution, meta-heuristic algorithms are extremely helpful in this situation. In this connection, problems (4), (8), and (12) are numerically solved with the help of the Teaching–Learning-Based Optimizer Algorithm (TLBOA) (Rao et al.^64^) due to its effectiveness. Furthermore, other two well-known algorithms, viz., the Grey Wolf Optimizer Algorithm (GWOA) (Mirjalili et al.^65^) and the Whale Optimizer Algorithm (WOA) (Mirjalili and Lewis^66^), are applied to compare the results of TLBOA.

### Brief description of teaching–learning-based optimizer algorithm (TLBOA)

Based on the processes of teaching and learning, Teaching Learning Based Optimisation (TLBO) was created. First of all, this idea was first presented by Rao et al.^64^ in 2011. The lesson that the students’ lecturer delivers in the classroom is beneficial to them. Teachers are continually encouraged to increase their pupils’ participation in class. Therefore, each student tries to adhere to the teacher’s directions in order to raise the performance of his or her group. However, every student has the chance to connect with other students in the classroom and develop their own talents. These served as the TLBO algorithm’s primary sources of inspiration. The teacher-student phase and the learner phase are thus the two phases of the TLBO algorithm.

#### Initialization

The TLBO algorithm begins with an initiation step, just like any other random search method. At the beginning of this technique, *L* initial solutions are randomly generated. As a result, during initialization, *L* random vectors of size *D* are created. As a result, a matrix with *D* columns and *L* rows is randomly constructed within the search space. The vector known as the learner represents one possible solution in the search space*. L* is the population size, sometimes known as the ‘class size’. The value *D*, which is the same as the problem’s dimensionality, is used to indicate how many ‘subjects or courses provided’ are available. In the search space, a *D*-dimensional vector represents each learner. Due to the iterative nature of the algorithms, the TLBO is programmed to run for *G* generations. The following equation is used in the first generation to determine the values for the *j*-th component of the *i*-th vector (learner).

$$U_{(i,j)}^{(0)} = U_{j}^{\min } + rand_{{\left( {i,j} \right)}} \times \left( {U_{j}^{\max } - U_{j}^{\min } } \right)$$

where $rand_{{\left( {i,j} \right)}}$ is the random variable and it is distributed uniformly over $(0,1)$. The *i*-th vector (or learner) for the *k*-th generation is given by,

$$U_{(i)}^{(k)} = \left[ {u_{(i,1)}^{(k)} ,u_{(i,2)}^{(k)} ,...,u_{(i,j)}^{(k)} ,...,u_{(i,D)}^{(k)} } \right]$$

#### Phase of teaching

A set of *L* learners who were chosen at random to start the assessment process are used to test the explore strategy. After an exam is given inside the classroom, each student is assessed. The teacher chooses the best pupils from the group of students that were tested. The teacher either imparts his or her knowledge to the other students in the class or encourages them to improve their own performance in the classroom. In order to improve each learner’s performance, teachers and students shared knowledge throughout this process. As a result, a teacher enhances the class's general fitness to the best of his or her ability. The following mathematical representation of this idea is provided:

The mean vector $\mu_{g}$ for each individual treated is computed at the *k*-th generation as,

$$\mu_{(k)} = \left[ {\sum\limits_{i = 1}^{L} {\left( {\frac{{u_{(i,1)}^{(k)} }}{L}} \right),\,\sum\limits_{i = 1}^{L} {\left( {\frac{{u_{(i,2)}^{(k)} }}{L}} \right),...,\sum\limits_{i = 1}^{L} {\left( {\frac{{u_{(i,j)}^{(k)} }}{L}} \right),...,\sum\limits_{i = 1}^{L} {\left( {\frac{{u_{(i,D)}^{(k)} }}{L}} \right)} } } } } \right],$$

where $$\mu_{(k)} = \left[ {m_{1}^{(k)} ,m_{2}^{(k)} ,...,m_{j}^{(k)} ,...,m_{D}^{(k)} } \right]$$.

The instructor $\left( {U_{teacher}^{k} } \right)$ is the most efficient vector and the greatest value of the objective function for that iteration (let us say the *k*-th). The algorithm moves the means of the other students in the direction of the instructor because he or she has the best mean among the group of students. The weighted differential vector and the present mean and target mean vectors are randomly merged, and this new population of improved learners is then added to the existing student population by the following equation:

$$Unew_{(i)}^{(k)} = U_{(i)}^{(k)} + rand^{(k)} \times \left( {U_{teacher}^{(k)} - T_{f} \mu_{(k)} } \right)$$

For each iteration, $T_{f}$ is a randomly chosen teaching factor that will be either 1 or 2. As a result, the more proficient learners in the population replace the less proficient ones.

#### Learning phase

A learner can learn in the classroom in two different ways. In order to raise their own learning levels, the learners interact with both the teacher and the other students. As a result, during this period, students interact in a group setting. The learner’s understanding typically grows as a result of this reciprocal engagement technique. Since each student interacts with other students at random, knowledge sharing is encouraged. Each participant is paired with a unique learner, and $U_{(i)}^{(g)}$ is selected at random $\left( {i \ne j} \right)$. In the learner phase, the *i*-th vector of the matrix $Unew$ is:

$$Unew_{(i)}^{(g)} = \left\{ {\begin{array}{l} {U_{(i)}^{(g)} + rand_{(i)}^{(g)} \times \left( {U_{(i)}^{(g)} - U_{(i)}^{(r)} } \right),\,\,if\,\left( {V_{(i)}^{(g)} < V_{(r)}^{(g)} } \right)} \\ {U_{(i)}^{(g)} + rand_{(i)}^{(g)} \times \left( {U_{(i)}^{(g)} - U_{(i)}^{(r)} } \right),\,otherwise} \\ \end{array} } \right.$$

#### Criteria of termination

The *k*-th iteration of this method ends with the reporting of the best result.

### Ethical approval

This article does not contain any human or animal participation by any of the authors.

## Numerical illustration

The objective functions $\, AP_{1} \left( {L,p,g,T} \right)$, $\, AP_{2} \left( {L,p,g,T} \right)$, and $\, AP_{3} \left( {L,p,g,T} \right)$ related to the maximization problems (4), (8), and (12) are highly nonlinear in nature with respect to the selling price of the product, green level of the product, payment period and cycle length. Therefore, it is a very difficult task to solve the problems (4), (8), and (12) by analytical methods. Due to the highly nonlinear objective functions, three numerical examples are considered to find the best-found solutions numerically.

### Numerical examples

Due to the consideration of trade credit period $L \ge 0$, two subcases arise, one for $L > 0$ and another for $L = 0$. So, there are a total of three examples considered to illustrate the proposed model. Example 1 and Example 2 are considered for Case-I and Case-II, respectively, whereas Example 3 is taken for Case-III.

#### Example 1

The following values of parameters are considered for Case-I.

$$K = 100$$, $$C_{1} = \$ 20$$, $$C_{2} = \$ 0.25$$, $$d_{1} = 0.1$$, $$C_{h} = \$ 2$$, $$C_{o} = \$ 30$$, $$r = \$ 0.02$$, $$\alpha = 1.2$$, $$a = 1.2$$, $$\lambda = 0.5$$, $$b = 1.5$$, $$\gamma = 0.8$$, $$c = 0.85$$, $$\xi = 1.2$$.

The best-found and worst-found results for Example 1 obtained from different algorithms, including TLBOA, GWOA and WOA, are provided in Tables 1 and 2, respectively. Also, statistical results are presented in Table 3.

**Table 1:** Best-found solution obtained from different algorithms in Case-I for Example 1.

Algorithms	$$AP_{1} \left( {L,p,g,T} \right)$$($/year)	$$L$$(year)	$$p$$($/unit)	$$T$$(year)	$$g$$	$$Run\,time$$(second)
TLBOA	680.229109	− 0.267182	39.594430	0.858030	0.933739	0.741076
GWOA	680.229104	− 0.267384	39.594517	0.858265	0.934152	0.280026
WOA	680.215410	− 0.258647	39.555326	0.859640	0.859640	0.209736

**Table 2:** Worst-found solution obtained from different algorithms in Case-I for Example 1.

Algorithms	$$AP_{1} \left( {L,p,g,T} \right)$$($/year)	$$L$$(year)	$$p$$($/unit)	$$T$$(year)	$$g$$	$$Run\,time$$(second)
TLBOA	680.229109	− 0.267182	39.59443	0.85803	0.933739	0.875044
GWOA	679.542987	− 0.002138	38.609008	0.858725	0.926515	0.272357
WOA	680.014294	− 0.40096	40.169053	0.823662	0.823662	0.280386

**Table 3:** Results of statistical experiment obtained from different algorithms in Case-I for Example 1.

Algorithm	Best-found $AP_{1}$	Worst-found $AP_{1}$	Mean of $AP_{1}$	Mode of $AP_{1}$	Median of $AP_{1}$	Standard deviation
TLBOA	680.229109	680.229109	680.229109	680.229109	680.229109	0
GWOA	680.229104	679.542987	680.215324	680.229088	680.229067	0.097023
WOA	680.215410	680.014294	680.195596	–	680.208660	0.033446

#### Example 2

The following values of parameters are considered for Case-II.

$$K = 100$$, $$C_{1} = \$ 20$$, $$C_{2} = \$ 0.25$$, $$d_{1} = 0.1$$, $$C_{h} = \$ 2$$, $$C_{o} = \$ 30$$, $$r = \$ 0.02$$, $$\alpha = 5.5$$, $$a = 0.3$$, $$\lambda = 0.5$$, $$b = 1.3$$, $$\gamma = 0.8$$, $$c = 0.7$$, $$\xi = 1.2$$, $$L = 0.$$

Tables 4 and 5 present the results of the best- and worst-found searches in Case-II for Example 2, which were conducted using various algorithms such as TLBOA, GWOA, and WOA. Additionally, Table 6 presents the statistical data.

**Table 4:** Best-found solution obtained from different algorithms in Case-II for Example 2.

Algorithms	$$AP_{2} \left( {L,p,g,T} \right)$$($/year)	$$L$$(year)	$$p$$($/unit)	$$T$$(year)	$$g$$	$$Run\,time$$(second)
TLBOA	727.917503	0	40.732595	0.879209	0.891952	0.997061
GWOA	727.917503	0	40.732213	0.879251	0.891763	0.557118
WOA	727.917500	0	40.732661	0.879451	0.89221	0.234018

**Table 5:** Worst-found solution obtained from different algorithms in Case-II for Example 2.

Algorithms	$$AP_{2} \left( {L,p,g,T} \right)$$($/year)	$$L$$(year)	$$p$$($/unit)	$$T$$(year)	$$g$$	$$Run\,time$$(second)
TLBOA	727.917503	0	40.732595	0.879209	0.891952	1.371319
GWOA	727.917411	0	40.733296	0.88038	0.895073	0.635222
WOA	223.869700	0	40.733296	0.88038	0.895073	0.635222

**Table 6:** Results of statistical experiment obtained from different algorithms in Case-II for Example 2.

Algorithm	Best-found $AP_{2}$	Worst-found $AP_{2}$	Mean of $AP_{2}$	Mode of $AP_{2}$	Median of $AP_{2}$	Standard deviation
TLBOA	727.917503	727.917503	727.917503	727.917503	727.917503	0
GWOA	727.917503	727.917411	727.9174842	727.917497	727.917489	$1.86 \times {10}^{ - 15}$
WOA	727.9175	727.671594	727.8962194	727.917435	727.9144995	0.049073829

#### Example 3

The following values of parameters are considered for Case-III.

$$K = 100$$, $$C_{1} = \$ 20$$, $$C_{2} = \$ 0.25$$, $$d_{2} = 0.{0005}$$, $$C_{h} = \$ 2$$, $$C_{o} = \$ 130$$, $$r = \$ 0.02$$, $$\alpha = 0.9$$, $$a = 0.3$$, $$\lambda = 0.5$$, $$b = 1.3$$, $$\gamma = 0.8$$, $$c = 0.75$$, $$\xi = 1.3$$.

Tables 7 and 8 present the best-found and worst-found outcomes achieved by the use of several algorithms (TLBOA, GWOA, and WOA) in Case-III for Example 3. Furthermore, the statistical findings are displayed in Table 9.

**Table 7:** Best-found solution obtained from different algorithms in Case-IIII for Example 3.

Algorithms	$$AP_{3} \left( {L,p,g,T} \right)$$($/year)	$$L$$(year)	$$p$$($/unit)	$$T$$(year)	$$g$$	$$Run\,time$$(second)
TLBOA	661.885590	0.190114	41.325618	1.845585	0.873231	0.540560
GWOA	661.885576	0.190436	41.326122	1.846089	0.874462	0.466127
WOA	661.885509	0.190969	41.324823	1.845661	0.869273	0.182543

**Table 8:** Worst-found solution obtained from different algorithms in Case-III for Example 3.

Algorithms	$$AP_{3} \left( {L,p,g,T} \right)$$($/year)	$$L$$(year)	$$p$$($/unit)	$$T$$(year)	$$g$$	$$Run\,time$$(second)
TLBOA	661.885590	0.190114	41.325618	1.845585	0.873231	0.643815
GWOA	661.885315	0.193069	41.325225	1.846077	0.872953	0.465553
WOA	661.613262	0.270290	41.300249	1.827551	0.709637	0.197036

**Table 9:** Results of statistical experiment obtained from different algorithms in Case-III for Example 3.

Algorithm	Best-found $AP_{3}$	Worst-found $AP_{3}$	Mean of $AP_{3}$	Mode of $AP_{3}$	Median of $AP_{3}$	Standard deviation
TLBOA	661.885590	661.885590	661.885590	661.885590	661.885590	$8.03 \times {10}^{ - 13}$
GWOA	661.885576	661.885315	661.8855147	661.885556	661.8855405	$6.49 \times {10}^{ - 5}$
WOA	661.885509	661.613262	661.8564689	–	661.8714095	0.0486201

Based on the computational results presented above, the following observations are derived:

(i) Based on the data shown in Table 1, it is observed that the TLBOA algorithm yields the best-found solution for Case-I in Example 1, whereas the solution generated from the WOA algorithm is the least. Moreover, it is shown from Tables 1 and 2 that the best-found and worst-found solutions obtained using the TLBOA algorithm are identical up to six decimal places. Moreover, WOA demonstrates a notable advantage in terms of computational efficiency since it requires little computing time to identify the best-found solution.
(ii) Table 3 presents the statistical measures of the mean, mode, median, and standard deviation acquired by the TLBOA algorithm in comparison to two other algorithms. The TLBOA method consistently yields the highest values for the mean, mode, and median. Additionally, it demonstrates the lowest standard deviation for Case-I in Example 1.
(iii) Based on the findings shown in Tables 4 and 7, it is inferred that the TLBOA yields the highest maximum solution for Case-II and Case-III in Examples 2 and 3. Furthermore, it is seen that the best-found solution and worst-found solution provided by the TLBOA are identical up to six decimal places.
(iv) In the context of determining the best-found solution, WOA demonstrates superior computational efficiency compared to TLBOA and WOA in Case-II and Case-III for Examples 1 and 2.
(v) In accordance with the data shown in Tables 6 and 7, it is apparent that the standard deviation under TLBOA is the lowest. Additionally, the mean, mode, and median values are identical up to six decimal places for Case II and III in Examples 2 and 3, with the exception of GWOA and WOA.

**Figure 3:**
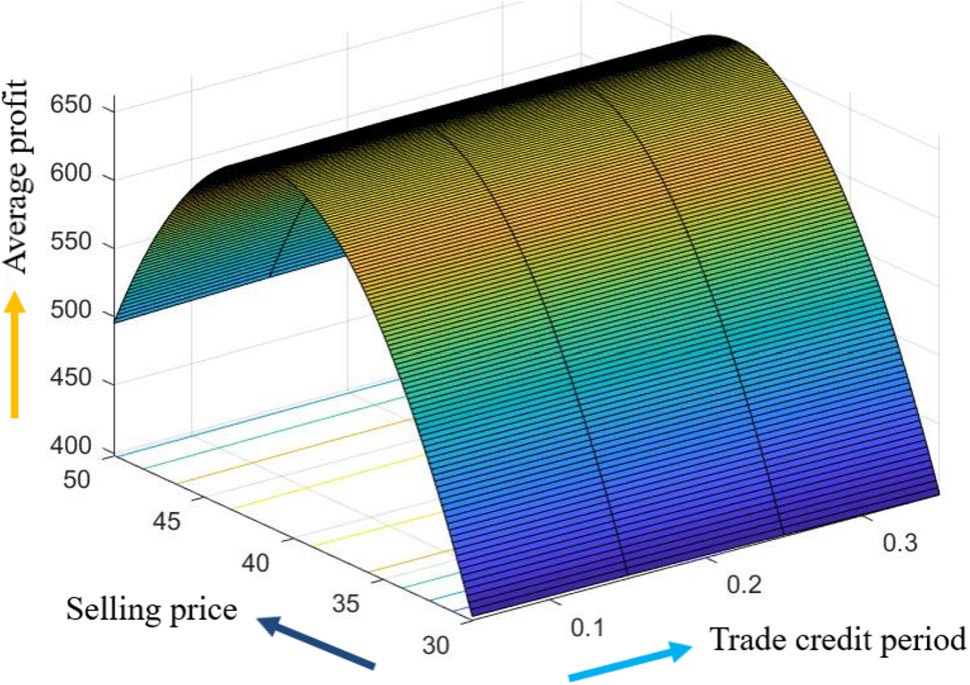
Concavity of average profit w.r.t. selling price and credit period of Example 3.

**Figure 4:**
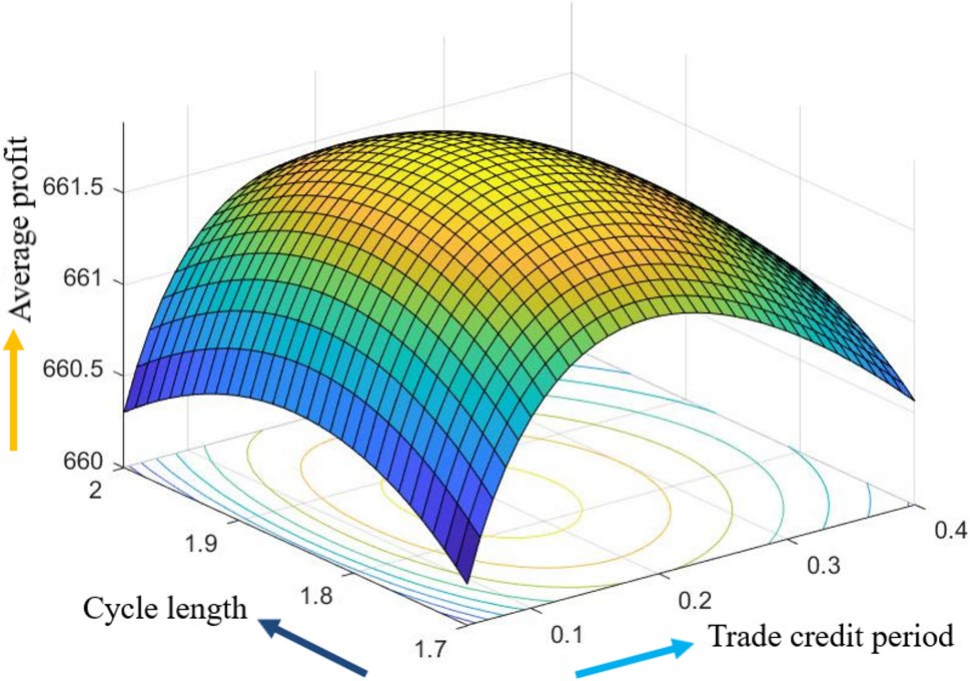
Concavity average profit w.r.t. credit period and cycle length of Example 3.

**Figure 5:**
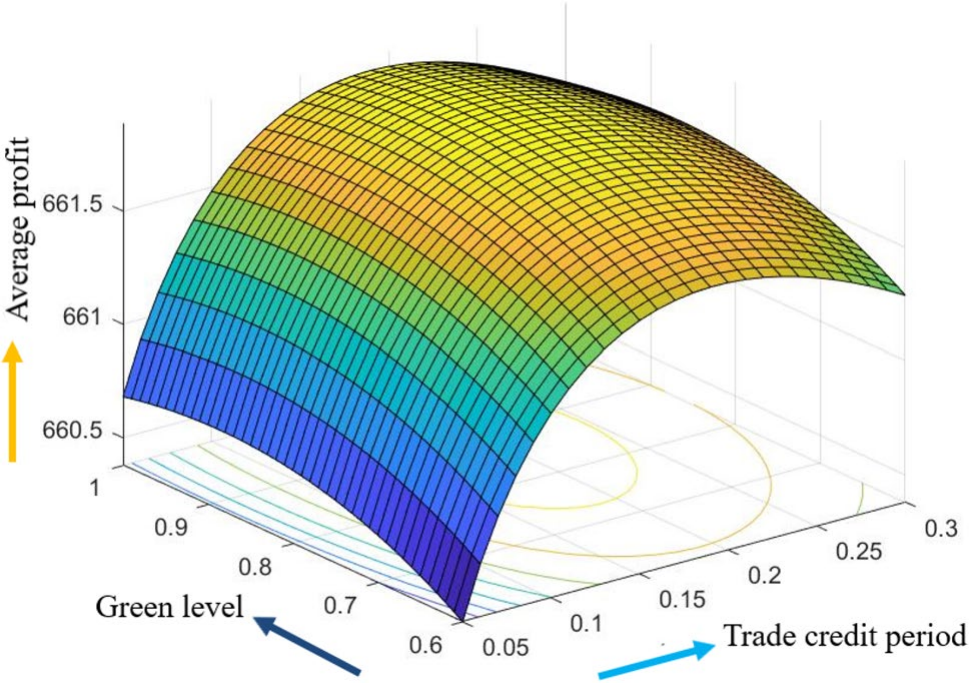
Concavity of average profit w.r.t. credit period and green level of Example 3.

**Figure 6:**
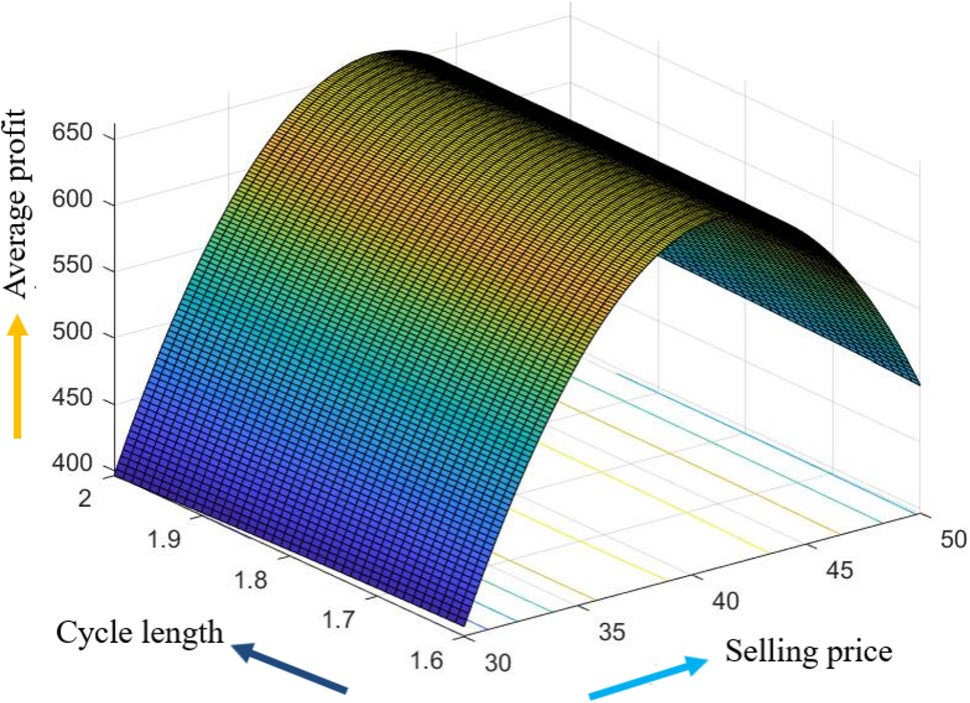
Concavity of average profit w.r.t. selling price and cycle length of Example 3.

**Figure 7:**
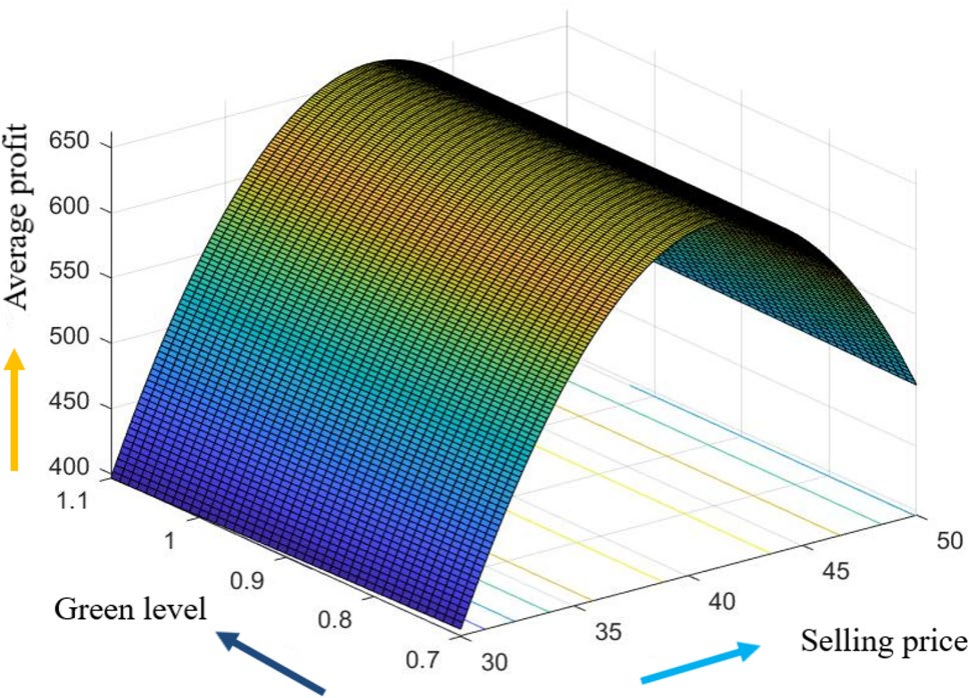
Concavity of average profit w.r.t. selling price and green level of Example 3.

**Figure 8:**
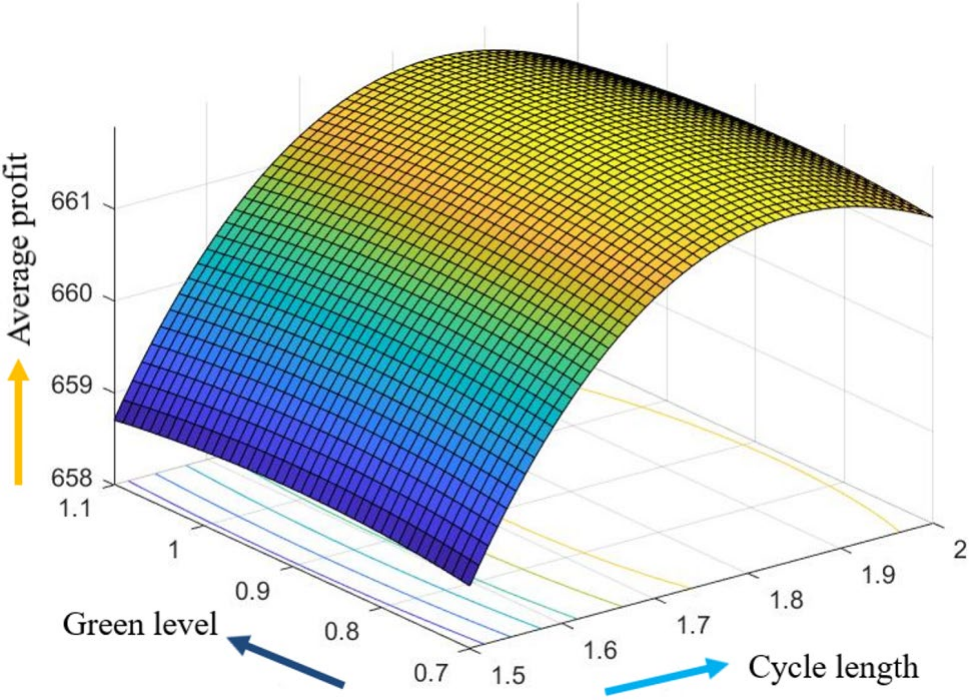
Concavity of average profit w.r.t. cycle length and green level of Example 3.

The graphical representation of the concavity of average profit $AP_{3} \left( {L,p,g,T} \right)$ with regard to various decision variables is depicted in Figs. 3, 4, 5, 6, 7, 8, utilizing the MATLAB software.

### ANOVA test

To analyze the significance of all the solutions obtained from TLBOA, GWOA, and WOA in Case-I for Example 1, an analysis of variance (ANOVA) is performed. At the time of this experiment, TLBOA is considered a controlling algorithm. The statistical solution of ANOVA is presented in Table 10.

**Table 10:** Analysis of variance (ANOVA) in Case-I of Example 1.

TLBOA vs	Count	Average	Variance	Source of variation						F	P-value	F-crit
				Between groups			Within group					
				SS	df	MS	SS	df	MS			
GWOA	50	661.88551	$4.21 \times 10^{ - 9}$	$1.41 \times 10^{ - 7}$	1	$1.41 \times 10^{ - 7}$	$2.06 \times 10^{ - 7}$	98	$2.10 \times 10^{ - 9}$	67.37	${\mathbf{9}}{\mathbf{.11 \times 10}}^{{{\mathbf{ - 13}}}}$	3.93
WOA	50	661.85648	0.002363	0.021200	1	0.021200	0.115832	98	0.001181	17.93	${\mathbf{5}}{\mathbf{.15 \times 10}}^{{{\mathbf{ - 5}}}}$	3.93

Significant values are in bold.

Table 10 reveals that the F statistical value for each method surpasses the F-critical value for Case-I in Example 1. Furthermore, it is noted that the p-value for both GWOA and WOA is lower than the predetermined significance level (0.05). In this particular instance, the null hypothesis is rejected.

### Convergence graph

**Figure 9:**
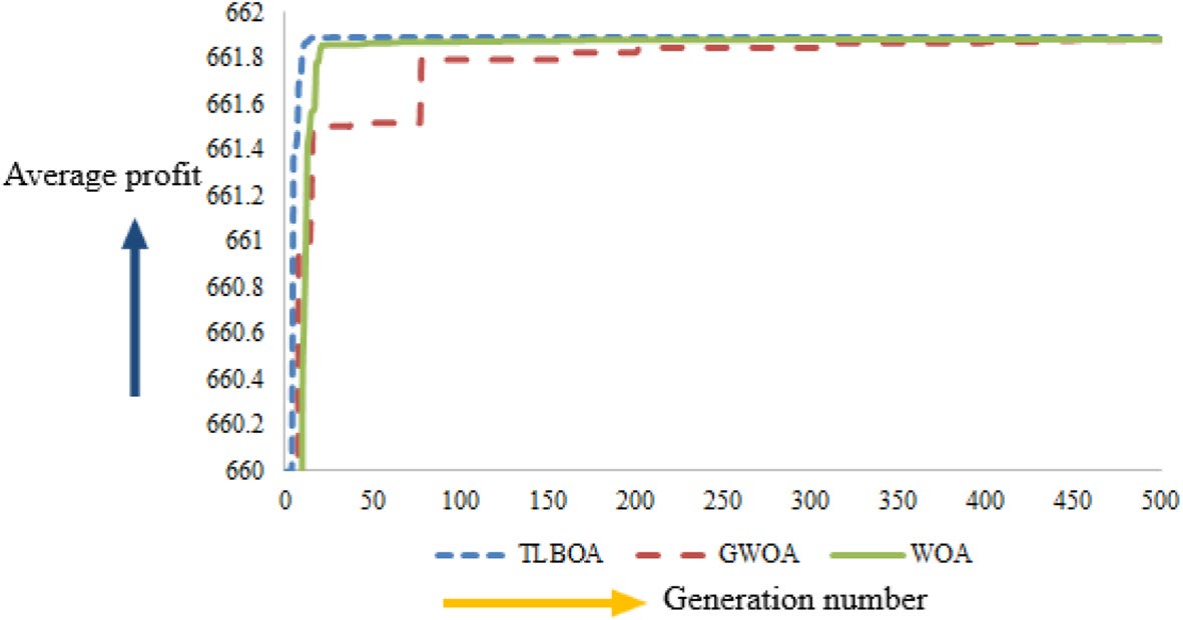
Convergence history of TLBOA, GWOA, and WOA in Case-I of Example 1.

The convergence rates of all the metaheuristic algorithms (TLBOA, GWOA, and WOA) are presented in Fig. 9.

From Fig. 9, it is observed that TLBOA converges to the best-found solution of the objective function after a minimum number of generations, whereas GWOA takes the maximum number of generations to converge to the best-found value.

## Sensitivity analyses

In this section, sensitivity analyses are performed to analyze the effects of best-found values of $AP_{3} \left( {L,p,g,T} \right)$, $L$, $p$, $T$, and $\theta_{g}$ with respect to different inventory parameters of Case-III for Example 3. This experiment is made by changing one parameter from − 20 to + 20% while keeping other parameters fixed. The sensitivity analysis of the respective parameter is shown graphically in Figs. 10, 11, 12, 13, 14, 15, 16, 17, 18, 19, 20, 21, 22, 23.

**Figure 10:**
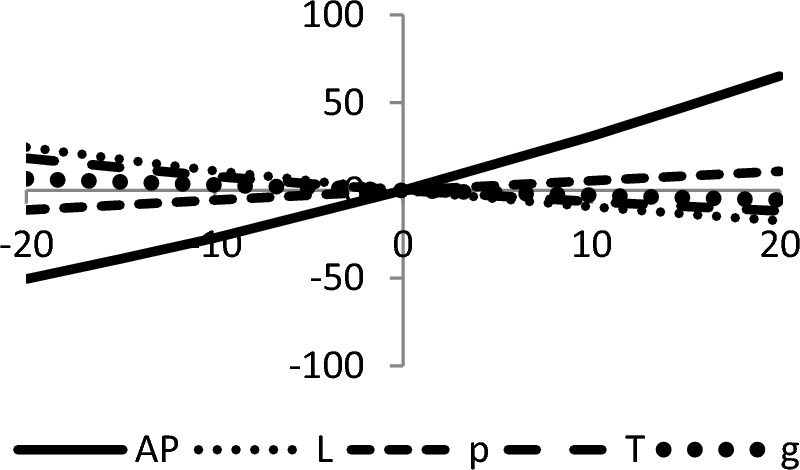
Effects on optimal policy of $K$.

**Figure 11:**
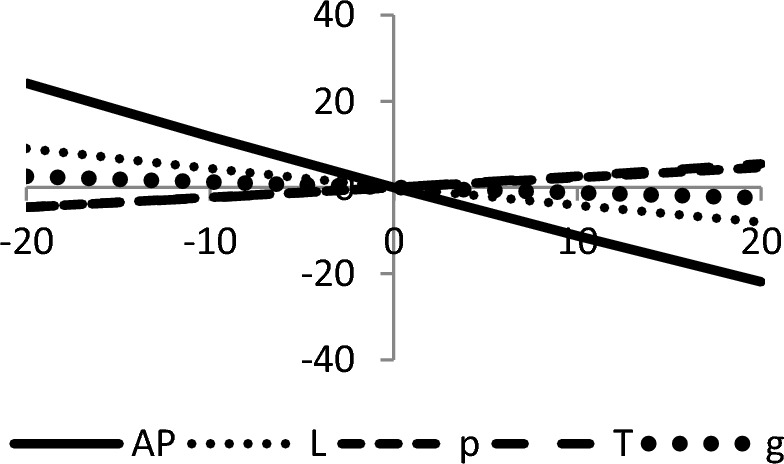
Effects on optimal policy of $C_{1}$.

**Figure 12:**
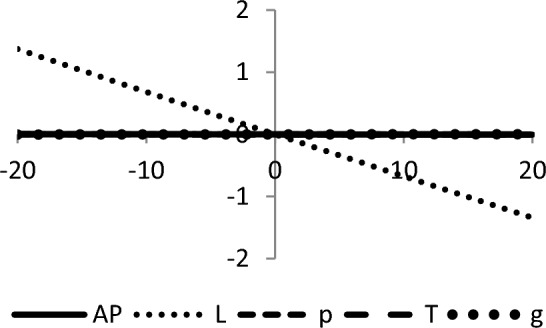
Effects on optimal policy of $d_{2}$.

**Figure 13:**
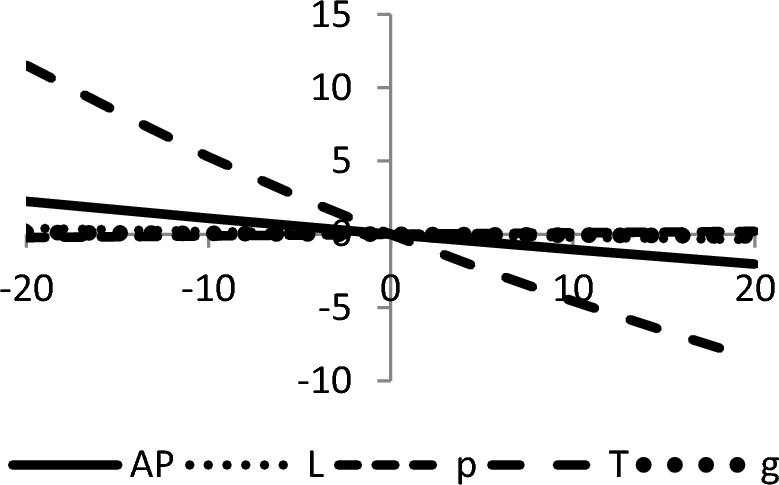
Effects on optimal policy of $C_{h}$.

**Figure 14:**
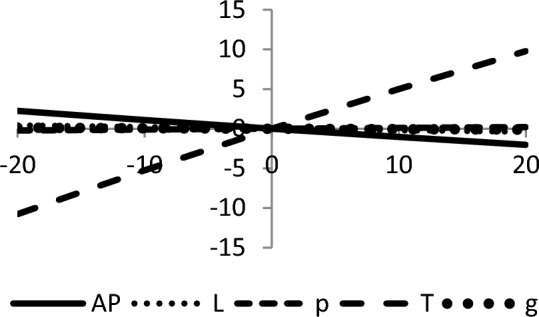
Effects on optimal policy of $C_{o}$.

**Figure 15:**
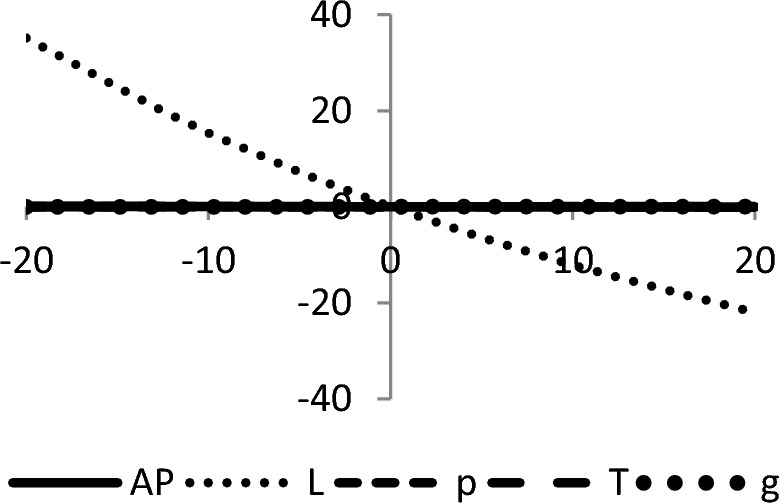
Effects on optimal policy of $r$.

**Figure 16:**
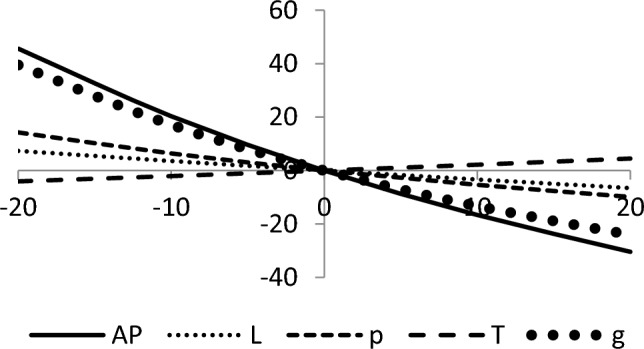
Effects on optimal policy of $\lambda$.

**Figure 17:**
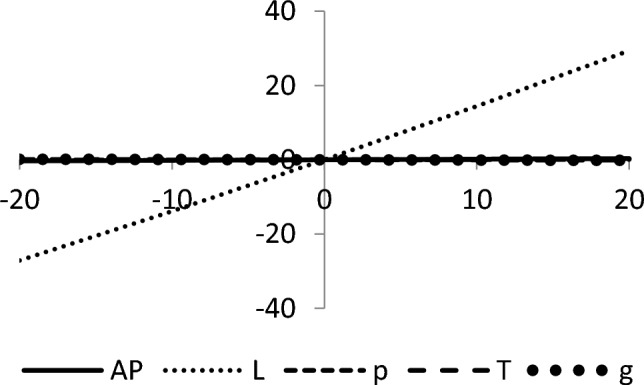
Effects on optimal policy of $\alpha$.

**Figure 18:**
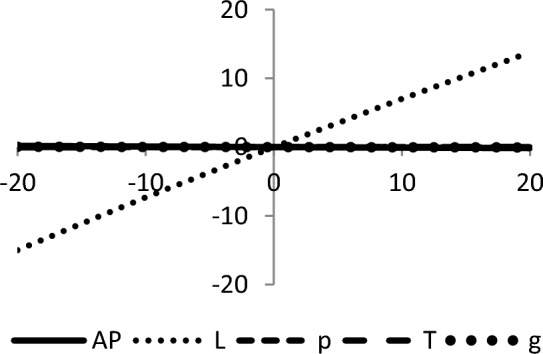
Effects on optimal policy of $a$.

**Figure 19:**
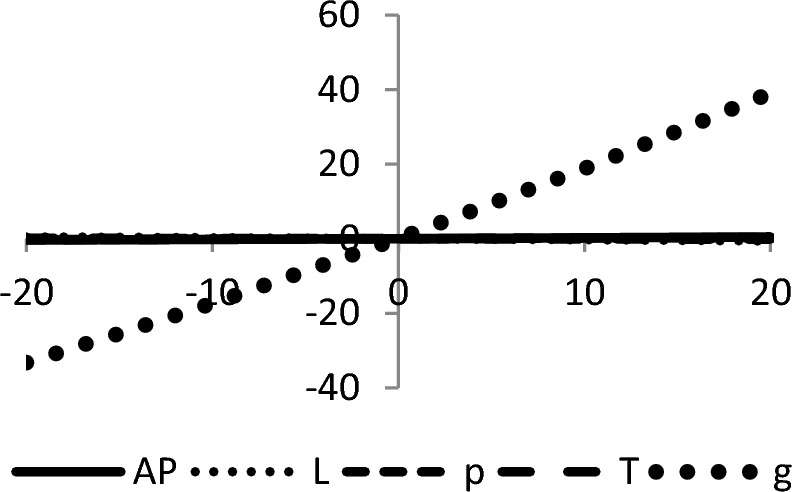
Effects on optimal policy of $\gamma$.

**Figure 20:**
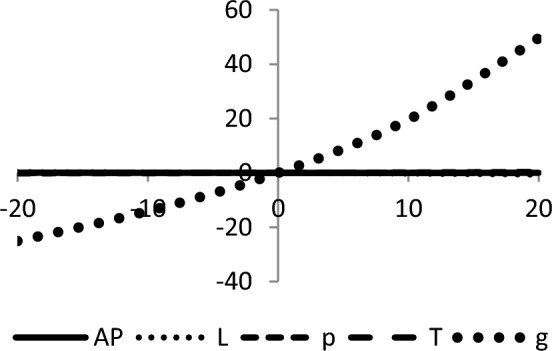
Effects on optimal policy of $c$.

**Figure 21:**
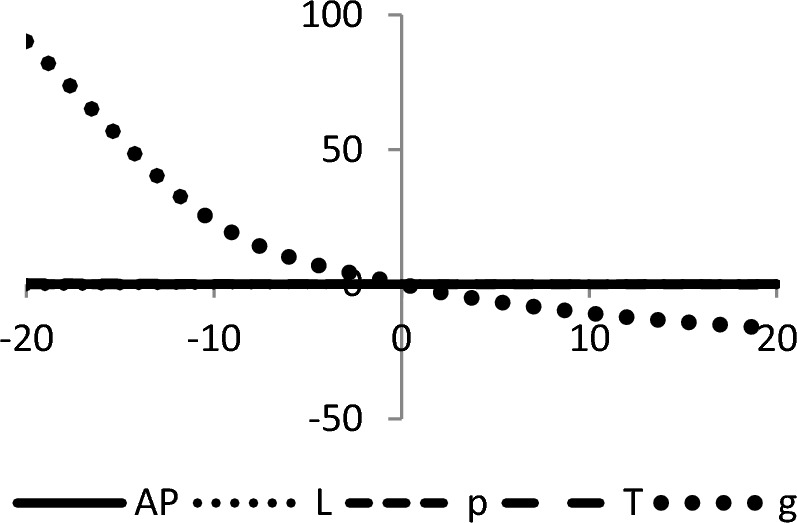
Effects on optimal policy of $\xi$.

**Figure 22:**
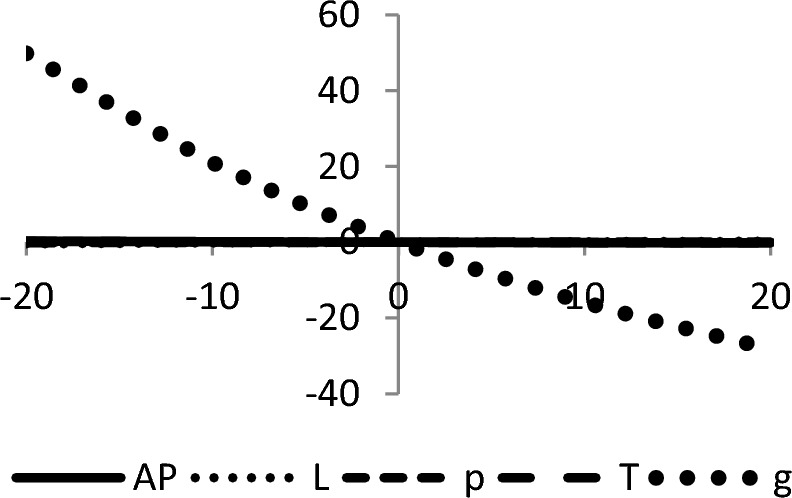
Effects on optimal policy of $C_{2}$.

**Figure 23:**
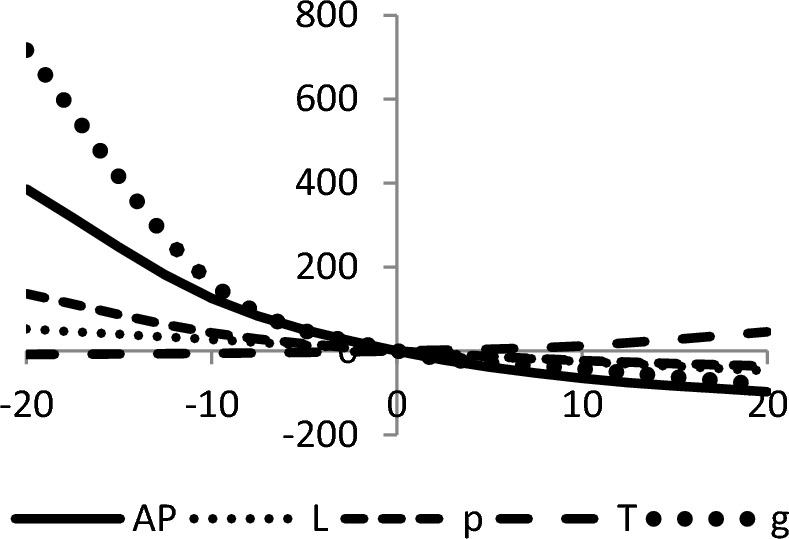
Effects on optimal policy of $b$.

From the sensitivity figure, the following observations are made:

From Fig. 10**,** it is clear that an inverse correlation exists between the percentage change in the demand parameter $K$ and the resulting profit. As the magnitude of the percentage change in $K$ grows, ranging from − 20 to 20%, there is a corresponding loss in profit. This observation suggests that a decrease in $K$ leads to an increase in profits, whereas an increase in $K$ leads to a decrease in earnings. Moreover, the percentage change in $K$ and the payment period have a positive correlation. As the magnitude of the percentage change in $K$ grows, there is a corresponding increase in the duration of the payment term. This implies that a reduction in $K$ is associated with an extension of payment durations, while an increase in $K$ results in a contraction of the payment time. In addition, an increasing $K$ leads to higher selling prices. The percentage change in $K$ and the green level have a positive correlation. As the percentage change in $K$ increases, there is a corresponding increase in the green level. This implies that there is a positive correlation between higher $K$ values and the adoption of environmentally sustainable practices or the production of eco-friendly items.

Figure 11 shows that trade credit period $L$, selling price $p$, cycle length $T$ and level of greenness of the product $g$ are less sensible, also profit per unit $AP_{3}$ is highly responsive with the same percentage change of $C_{1}$. Reducing purchasing costs related to $C_{1}$ leads to higher profits, while increasing these costs results in lower profitability. As $C_{1}$ cost parameter decreases, the selling price tends to decrease, and as $C_{1}$ cost increases, the selling price increases. This indicates that controlling purchasing cost can allow businesses to set lower selling prices, which leads to higher profits for the seller.

Figure 12 illustrates the effects of altering the default risk parameter ($d_{2}$) on several significant financial and operational key performance indicators for the seller. The fluctuation in profit, payment period, selling price, cycle duration, and green level remains modest when the default risk parameter ($d_{2}$) is altered by ± 10% and ± 20%. This implies that, within the given range of alterations in default risk parameters, the financial and operational performance of the firm is reasonably consistent. This observation suggests that the organization has implemented robust risk management strategies to minimize the potential consequences arising from variations in the default risk parameter.

According to the data presented in Fig. 13, it is observed that a drop in the holding cost parameter is associated with a minor gain in profit. Additionally, the payment period exhibits a rather consistent trend, while the selling price demonstrates a decline. Concurrently, there is a slight decrease in the duration of the cycle period, although the degree of green stays generally consistent. The results of this study indicate that the reduction of the holding cost parameter leads to increased profitability and could potentially affect pricing strategies, all while ensuring operational and environmental stability. These findings offer valuable insights for businesses aiming to strike a balance between cost-effectiveness and environmental sustainability.

The impact of varying the ordering cost ($C_{o}$) by ± 10% and ± 20% on several key financial and operational metrics is illustrated in Fig. 14. A reduction in the cost associated with placing the order leads to a moderate benefit in profits, a somewhat consistent payment period, and a drop in the selling price at which goods are sold. Concurrently, there is a minor drop in the cycle period, but the degree of green stays rather consistent. The results of this study indicate that by optimizing ordering costs, organizations may potentially enhance profitability and impact pricing strategies, all while ensuring operational and environmental stability.

Figure 15 shows that as the interest rate decreases, profit increases, payment periods remain stable, and selling prices also remain relatively constant. The cycle period experiences a slight increase, while the green level maintains its stability. In addition, Fig. 16 reveals that as the selling price controlling scale parameter in demand ($\lambda$) increases, profit decreases, the credit period remains relatively constant, and the selling price significantly increases. Simultaneously, the cycle period and green level are both negatively impacted. These findings suggest that changes in the selling price controlling scale parameter can have a substantial impact on profit and pricing strategies, potentially affecting operational efficiency and environmental considerations, providing valuable insights for businesses seeking to optimize their pricing while managing sustainability concerns. Furthermore, Figs. 17 and 18 indicate that the trade credit period $L$ is highly responsive, whereas the rest of the matrices are not sensitive to changes in the scale parameter $(\alpha )$ of the payment period and index $(a)$ of the payment period in demand.

From Figs. 19, 20, 21 and 22 one can observe that the green level $(g)$ of the product is highly sensitive, whereas financial and remaining operational key performance indicators for the seller are not affected by changes in % change of $\gamma$, $c$, $\xi$ and $C_{2}$. Additionally, the green level $(g)$ of the sustainable product increases when $\gamma$ and $c$ increase; however, it decreases, when $\xi$ and $C_{2}$ increase.

Figure 23 shows that profit and the percentage change in the unit price index ($b$) exhibit a strong negative connection. As the unit price index ($b$) lowers within the range of -20% to 20%, there is an observed rise in profit. This implies that there is a positive correlation between decreasing the unit price index ($b$) and increasing profitability, whereas a rise in the unit price index ($b$) is related to a decrease in earnings. As the index $b$ decreases, the payment period significantly increases. Conversely, an increase in the unit price index ($b$) is associated with a shorter payment period. This indicates that controlling unit prices can influence the timing of payments. In addition, decreasing the unit price index ($b$) tends to lead to higher selling prices, while increasing the unit price index ($b$) results in lower selling prices. Reducing the index $b$ is associated with a higher green level, while increasing the unit price index ($b$) results in a lower green level.

## Managerial insights

The implementation of statistical techniques in the field of management provides unique insights that hold considerable significance for policymakers. The following are management insights pertaining to the determination of selling price, green level, payment period, and replenishment cycle length for enterprises engaged in the trade of sustainable green products.

- The seller’s strategy should involve selling items with a higher green level when the index of the green level in procurement costs decreases. The increasing acceptance of sustainable product offerings, driven by the declining costs of environmentally friendly sourcing, presents opportunities for both ecological benefits and economic savings.
- With an increase in the index of the green level in demand, there is a noticeable surge in consumers’ demand for sustainable green commodities. Therefore, it is advisable for the seller to prioritize the trade of sustainable green commodities that have higher green levels.
- The identified positive correlation between the change in ordering cost and profit suggests that organizations can improve their profitability by implementing cost reduction initiatives in their supply chain and procurement operations. Companies have the opportunity to boost profit margins through the reduction of expenses related to order placement. This observation emphasizes the importance of consistently seeking ways to optimize procurement and supply chain activities to enhance overall financial performance.
- When ordering costs decrease, companies can negotiate extended payment terms, facilitating more efficient cash flow management. Conversely, when ordering expenses increase, organizations can negotiate shorter payment terms.
- The identified negative correlation between the change in the unit price index ($b$) and profit suggests that organizations could potentially enhance their profitability through strategic management and a reduction in unit prices. Implementing cost-saving initiatives within procurement and supply chain operations has the potential to lead to increased profitability. This observation underscores the crucial significance of cost management in the pursuit of maximizing profits.
- In cases of rising interest rates, it is advisable for the company to contemplate offering a shorter loan duration. This strategy is designed to alleviate the financial impact stemming from potential losses due to delayed payments and increased default risk.

## Conclusions

Even though there are very few studies on the topic of pricing and lot-sizing decisions for sustainable green products in the context of credit payments, the majority of studies have predominantly focused on the perspective of the buyer. To the best of the authors’ knowledge, this is the first study accomplished by adopting the consequences of prepayment duration on the demand rate for green products and tries to achieve the best inventory management strategies for a seller dealing with sustainable green products by considering the consequences of payment period, selling price, and green level on the consumption rate of these items. The seller’s average profit within a singular business cycle is established by the implementation of three distinct payment structures, namely the advance, cash, and credit payment options, where the average profit functions are highly nonlinear in nature. Consequently, the maximization problems are often addressed utilizing the widely recognized metaheuristic algorithm TLBOA, and the outcomes obtained from TLBOA are compared to those achieved via the use of GWOA and WOA. To provide management insights, sensitivity studies are conducted to analyze the influence of financially linked characteristics on the actions and earnings of the seller. When the index of green level in procuring costs decreases, the seller tries to sell products with a higher green level. The increasing adoption of sustainable product offerings, driven by the declining prices of green-level procurement, opens opportunities for both environmental advantages and financial savings. If the index of green level in demand increases, customers’ demand for sustainable green items increases significantly, and hence the seller tries to trade sustainable green commodities with higher green levels. Moreover, when the interest rate is high, the company should offer a lower credit period to reduce the interest loss from delay in payment and default risk. Finally, this study provides valuable insights for sustainable enterprises in determining the best pricing and level of environmental sustainability for their products by considering the procurement costs associated with the products’ green level attributes and their influence on customer demand.

There are several possible improvements that can be incorporated into this model. In recent years, several governments throughout the world have implemented charges on the carbon emission levels released by corporations. Therefore, a noteworthy addition to the current study would involve encompassing environmental emission regulations. In reality, consumer demand exhibits variability in several cases, contingent upon the quantity of stock available in storage. Consequently, the effects of the current stock level may be included in the demand function.

### Supplementary Information

Supplementary Information.

## Data Availability

The datasets used and/or analyzed during the current study are available from the corresponding author on reasonable request.
