# Supplementary material for: Inventory model for green products with payment strategy, selling price and green level dependent demand using teaching learning based optimization algorithm
Source: Sci Rep. 2024 Feb 6;14:3033. doi: 10.1038/s41598-024-53109-w (PMC10847503; doi:10.1038/s41598-024-53109-w)
Supplement: Supplementary file 1 — Supplementary Information. [file 41598_2024_53109_MOESM1_ESM.docx]

Supplementary material

Appendix A. Expressions of all $X_{i}$ ($i=1,2,...,11$)

$$\begin{matrix} X_{1}=2\left(d_{1}-\frac{1}{2}r-rd_{1}L\right)\left\{\alpha apL^{a-1}-\lambda bd_{1}\left(1+d_{1}L\right)^{b-1}p^{b+1}\right\} \\ +\left\{\left(1-\frac{1}{2}rL\right)\left(1+d_{1}L\right)p-\left(\frac{C_{h}T}{2}+C_{1}+C_{2}g^{\xi}\right)\right\}\left\{\alpha a(a-1)L^{a-2}-\lambda b(b-1)d_{1}^{2}\left(1+d_{1}L\right)^{b-2}p^{b}\right\}, \end{matrix}$$

$X_{2}=rd_{1}p\left\{K+\alpha L^{a}-\lambda\left\{\left(1+d_{1}L\right)p\right\}^{b}+\gamma g^{c}\right\}$,

$$\begin{matrix} X_{3}=\left(d_{1}-\frac{1}{2}r-rd_{1}L\right)\left\{K+\alpha L^{a}-\lambda\left\{\left(1+d_{1}L\right)p\right\}^{b}+\gamma g^{c}\right\}+\left(1-\frac{1}{2}rL\right)\left(1+d_{1}L\right)\left\{\alpha aL^{a-1}-\lambda bd_{1}\left(1+d_{1}L\right)^{b-1}p^{b}\right\} \\ +\frac{1}{2}r\left(1+d_{1}L\right)^{b+1}\lambda bp^{b}-\left(1-\frac{1}{2}rL\right)\lambda bp^{b}(b+1)d_{1}\left(1+d_{1}L\right)^{b}+\left(C_{h}\frac{T}{2}+C_{1}+C_{2}g^{\xi}\right)\lambda b^{2}d_{1}\left(1+d_{1}L\right)^{b-1}p^{b-1}, \end{matrix}$$

$X_{4}=\left(d_{1}-\frac{1}{2}r-rd_{1}L\right)p\gamma cg^{c-1}-C_{2}\xi g^{\xi-1}\left\{\alpha aL^{a-1}-\lambda bd_{1}p\left\{\left(1+d_{1}L\right)p\right\}^{b-1}\right\}$,

$X_{5}=\frac{C_{h}}{2}\left\{\alpha aL^{a-1}-\lambda bd_{1}p\left\{\left(1+d_{1}L\right)p\right\}^{b-1}\right\}$,

$X_{6}=-\left(1-\frac{1}{2}rL\right)\lambda b(b+1)\left(1+d_{1}L\right)^{b+1}p^{b-1}+\left(C_{h}\frac{T}{2}+C_{1}+C_{2}g^{\xi}\right)\lambda b(b-1)\left(1+d_{1}L\right)^{b}p^{b-2}$,

$X_{7}=\left(1-\frac{1}{2}rL\right)\left(1+d_{1}L\right)\gamma cg^{c-1}+C_{2}\xi g^{\xi-1}\lambda b\left(1+d_{1}L\right)^{b}p^{b-1}$, $X_{8}=\frac{C_{h}}{2}\lambda b\left(1+d_{1}L\right)^{b}p^{b-1}$,

$$\begin{matrix} X_{9}=\left(1-\frac{1}{2}rL\right)\left(1+d_{1}L\right)p\gamma c(c-1)g^{c-2}-c(c-1)\gamma g^{c-2}\left(C_{h}\frac{T}{2}+C_{1}+C_{2}g^{\xi}\right)-2C_{2}\xi c\gamma g^{c+\xi-2} \\ -C_{2}\xi(\xi-1)g^{\xi-2}\left\{K+\alpha L^{a}-\lambda\left\{\left(1+d_{1}L\right)p\right\}^{b}+\gamma g^{c}\right\}, \end{matrix}$$

$X_{10}=\frac{C_{h}}{2}c\gamma g^{c-1}$ , and $X_{11}=\frac{2C_{o}}{T^{3}}$.

Appendix B. Expressions of all $Y_{i}$ ($i=1,2,...,7$)

$Y_{1}=\left(\frac{1}{2}C_{h}T+C_{1}+C_{2}g^{\xi}\right)\lambda b(b-1)p^{b-2}$, $Y_{2}=\lambda b(b+1)p^{b-1}$, $Y_{3}=c\gamma g^{c-1}+C_{2}\lambda b\xi g^{\xi-1}p^{b-1}$, $Y_{4}=\frac{1}{2}C_{h}\lambda bp^{b-1}$, $Y_{5}=\left(p-\frac{1}{2}C_{h}T-C_{1}-C_{2}g^{\xi}\right)\gamma c(c-1)g^{c-2}-2C_{2}\gamma c\xi g^{c+\xi-2}-C_{2}\xi(\xi-1)g^{\xi-2}(K-\lambda p^{b}+\gamma g^{c})$, $Y_{6}=\frac{1}{2}C_{h}\gamma cg^{c-1}$, and $Y_{7}=\frac{2C_{o}}{T^{3}}$.

Appendix C. Expressions of all $Z_{i}$ ($i=1,2,...,11$)

$Z_{1}=p\alpha a(a-1)L^{a-2}e^{-d_{2}L}+d_{2}^{2}p(K+\alpha L^{a}-\lambda p^{b}+\gamma g^{c})e^{-d_{2}L}$, $Z_{2}=2d_{2}p\alpha aL^{a-1}e^{-d_{2}L}+\frac{1}{2}rp\alpha aL^{a-1}+\frac{1}{2}rp\alpha a^{2}L^{a-1} +\left(\frac{T}{2}C_{h}+C_{1}+C_{2}g^{\xi}\right)\alpha a(a-1)L^{a-2}$, $Z_{3}=\alpha aL^{a-1}e^{-d_{2}L}-\left(d_{2}e^{-d_{2}L}+\frac{1}{2}r\right)\{K+\alpha L^{a}-\lambda(b+1)p^{b}+\gamma g^{c}\}-\frac{1}{2}r\alpha aL^{a}$, $Z_{4}=\left(d_{2}e^{-d_{2}L}+\frac{1}{2}r\right)p\gamma cg^{c-1}+C_{2}\alpha a\xi g^{\xi-1}L^{a-1}$, $Z_{5}=\frac{1}{2}C_{h}\alpha aL^{a-1}$, $Z_{6}=\left(\frac{T}{2}C_{h}+C_{1}+C_{2}g^{\xi}\right)\lambda b(b-1)p^{b-2}-\lambda b(b+1)p^{b-1}\left(e^{-d_{2}L}-\frac{1}{2}rL\right)$, $Z_{7}=\gamma cg^{c-1}\left(e^{-d_{2}L}-\frac{1}{2}rL\right)+C_{2}\lambda b\xi g^{\xi-1}p^{b-1}$, $Z_{8}=\frac{1}{2}C_{h}\lambda bp^{b-1}$, $\begin{matrix} Z_{9}=\left(e^{-d_{2}L}-\frac{1}{2}rL\right)p\gamma c(c-1)g^{c-2}-2C_{2}\gamma c\xi g^{c+\xi-2}-\left(\frac{T}{2}C_{h}+C_{1}+C_{2}g^{\xi}\right)\gamma c(c-1)g^{c-2} \\ -C_{2}\xi(\xi-1)g^{\xi-2}(K+\alpha L^{a}-\lambda p^{b}+\gamma g^{c}), \end{matrix}$

$Z_{10}=\frac{1}{2}C_{h}\gamma cg^{c-1}$, and $Z_{11}=\frac{2C_{o}}{T^{3}}$.
